# Discovery of a novel ferroptosis inducer-talaroconvolutin A—killing colorectal cancer cells in vitro and in vivo

**DOI:** 10.1038/s41419-020-03194-2

**Published:** 2020-11-17

**Authors:** Yong Xia, Shuzhi Liu, Changlin Li, Zhiying Ai, Wenzhi Shen, Wenqi Ren, Xiaolong Yang

**Affiliations:** 1 Key Laboratory of Precision Oncology of Shandong Higher Education, Institute of Precision Medicine, Jining Medical University, 272067 Jining, Shandong P.R. China; 2 Departments of Urology, New York University School of Medicine, New York, NY 10016 USA; 3 The Modernization Engineering Technology Research Center of Ethnic Minority Medicine of Hubei Province, School of Pharmaceutical Sciences, South-Central University for Nationalities, 430074 Wuhan, P.R. China

**Keywords:** Colon cancer, Necroptosis, Small molecules

## Abstract

Ferropotsis is among the most important mechanisms of cancer suppression, which could be harnessed for cancer therapy. However, no natural small-molecule compounds with cancer inhibitory activity have been identified to date. In the present study, we reported the discovery of a novel ferroptosis inducer, talaroconvolutin A (TalaA), and the underlying molecular mechanism. We discovered that TalaA killed colorectal cancer cells in dose-dependent and time-dependent manners. Interestingly, TalaA did not induce apoptosis, but strongly triggered ferroptosis. Notably, TalaA was significantly more effective than erastin (a well-known ferroptosis inducer) in suppressing colorectal cancer cells via ferroptosis. We revealed a dual mechanism of TalaA’ action against cancer. On the one hand, TalaA considerably increased reactive oxygen species levels to a certain threshold, the exceeding of which induced ferroptosis. On the other hand, this compound downregulated the expression of the channel protein solute carrier family 7 member 11 (SLC7A11) but upregulated arachidonate lipoxygenase 3 (ALOXE3), promoting ferroptosis. Furthermore, in vivo experiments in mice evidenced that TalaA effectively suppressed the growth of xenografted colorectal cancer cells without obvious liver and kidney toxicities. The findings of this study indicated that TalaA could be a new potential powerful drug candidate for colorectal cancer therapy due to its outstanding ability to kill colorectal cancer cells via ferroptosis induction.

## Introduction

Colorectal cancer (CRC) is one of the most frequent cancer types. It is ranked the third most common morbidity and second global mortality, and associated with nearly two million new cases and ~900,000 deaths in 2018 alone^1,2^. Global statistics show that CRC is widespread, especially in economically developed regions, such as Europe, North America, Australia, and Japan^3^. In China, the incidence of CRC is on the rise with the continuous development of economy^3^. The occurrence of distal colon and rectal cancers has increased most rapidly in adolescents and young adults in recent years^4,5^. Various strategies have now been developed for the treatment of CRC, such as surgery, chemotherapy, radiotherapy, targeted therapy, and immunotherapy^6,7^. Chemoradiotherapy is often used before or after surgery to prevent the recurrence and metastases of the disease^8,9^. However, current chemotherapy drugs are not able to fully control CRC. Relapse occurs in ~30% of stages I–III and 65% of post-stage IV patients, which emphasizes the urgency of the search for new, more effective drugs^10^.

The cancer-preventive and anticancer activities of plant-derived small-molecule compounds, such as terpenoids, carotenoids, anthocyanidins, and flavonoids, have been extensively investigated^11^. Some compounds of the aforementioned small-compound classes regulate the gene expression, and are thus involved in crucial biological processes, such as cell proliferation, differentiation, apoptosis, and autophagy^6,12^. In addition to plant-derived small-molecule compounds, microbial-derived small-molecule compounds have also attracted substantial research attention in recent years, and have undergone screening for determination of their anticancer potential^13,14^. For example, Ekbatan et al. reported that chlorogenic acid suppressed the proliferation of colon cancer Caco-2 cells via cell cycle arrest and apoptosis induction. However, the IC_50_ value of chlorogenic acid for colon cancer cell proliferation was higher than 100 μM, which limited its feasibility^14^. Hence, no effective microbial molecules that are capable of destroying colon cancer cells have been identified. Therefore, we dedicated our efforts to search for new, more effective microbial sources of small-molecule compounds which can inhibit or kill CRC cells.

Cancer cell death can occur via different mechanisms, such as necrosis, apoptosis, autophagy, pyroptosis, and ferroptosis. Of them, ferroptosis is the most recently discovered cell death pathway that is based on the action of ferric ion and reactive oxygen species (ROS)^15^. Ferroptosis is morphologically and mechanistically distinct from apoptosis^16^. Cells undergoing ferroptosis have specific morphological features, including ruptured cell membranes, vesicle formation, reduced mitochondrial size, increased density of the mitochondrial membrane, decreased or disappeared mitochondrial ridge, and broken outer mitochondrial membrane; the nucleus has a normal size but lacks chromatin condensation^17^. An observation under an electron microscope reveals smaller-than-usual mitochondria and increased bilayer membrane density^18^. Emerging evidence suggests that ferroptosis is an ancient and delicate physiological process and that insufficient ferroptosis can induce carcinogenesis^19^. Ferropotsis is probably one of the most important mechanisms of cancer suppression, which could be harnessed for tumor therapy. The use of ferroptosis for the development of new anticancer strategies has recently attracted considerable research attention. In a previous study, cancer cells were found to have higher iron requirements than normal cells, a phenomenon known as “iron addiction”^20^. This specificity increases the susceptibility of cancer cells to lipid peroxidation-induced ferroptosis, which may provide new opportunities for cancer treatment^21^. Several small molecules and FDA-approved clinical drugs were established to promote ferroptosis in cancer cells. Therefore, the antitumor activities of ferroptosis inducers have been recently investigated in various experimental tumor models, which confirmed the potential of ferroptosis as a novel method of anticancer therapy^18,22^.

The anticancer activity of TalaA, a natural product isolated from the endophytic fungus *Talaromyces purpureogenus* inhabiting *Panax notoginseng*, has not been investigated until now. In our present study, multiple experimental perspectives were employed, and, as a result, we discovered that TalaA had the ability to kill various CRC cell lines (HCT116, SW480, and SW620). Furthermore, our in vivo experiment showed that TalaA suppressed the tumor growth in the transplant of xenograft nude mice.

In the present investigation, we found that TalaA did not induce apoptosis, but powerfully triggered ferroptosis by ROS upregulation, which led to lipid peroxidation and decreased the levels of antioxidant molecules. Morphologically, TalaA caused mitochondrial shrinkage and cell membrane perforation. Moreover, the ferroptosis inhibitor ferrostatin-1 neutralized the lethal effects of TalaA, which additionally verified that TalaA activated the ferroptosis pathway. On the other hand, transcriptome sequencing and enrichment analysis data showed that the ferroptosis pathway was among the major pathways in the KEGG-enrichment analysis results. We discovered that TalaA not only upregulated lipid peroxidases such as ALOXE3 and ALOX12, but also suppressed the synthesis of the antioxidant glutathione via downregulation of SLC7A11, a crucial channel protein involved in the transport of cystine from extracellular to intracellular sites. Besides, various iron metabolism-related genes, including *FTL*, *FTH1*, and *FTH1P23*, as well as *HMOX1* were also upregulated by TalaA treatment.

ROS is ubiquitous in living organisms^23^. It is not only a product of normal cell physiological activities, but also an important signaling molecule^24^. The growth rate and ROS levels in healthy cells of normal tissues are usually low. However, in cancer cells, ROS production is increased due to the vigorous cell metabolism and proliferation. Meanwhile, a set of antioxidant systems against ROS is derived by cancer cells to prevent themselves from damage caused by ROS; moreover, they can utilize ROS as a positive regulatory signal for advanced survival and proliferation^25^. When the oxidative stress in cells, caused by ROS, is too strong, they enter programmed death pathways, such as apoptosis and ferroptosis. Of note, cancer cells have higher baseline ROS levels than normal cells. Thus, a strategy to elevate the content of ROS and suppress the activities of antioxidant molecules, which induces cancer cell death, would be a highly sensible strategy for cancer treatment. Notably, in this study we found TalaA strongly elevated the ROS level in CRC cells, which was an important reason why TalaA killed cancer cells via ferroptosis. It is worth noting that the anticancer activity of TalaA is significantly higher than that of erastin in killing cancer cells and triggering ferroptosis. TalaA suppresses the growth of CRC cells through two pathways: (1) by elevation of cancer cell ROS level to initiate ferroptosis; (2) by alteration of the expression of ferroptosis-related molecules (e.g., SLC7A11, ALOXE3, GSS, and HMOX1), which accelerates ferroptosis. Due to its high anticancer activity and low toxicity, TalaA could be a powerful potential candidate drug for CRC chemotherapy. This study reveals the anticancer mechanism of TalaA, and provides important experimental evidence that will facilitate the development of novel anticancer drugs.

## Materials and methods

### Fermentation, extraction, and isolation

The fungus *T. purpureogenus* was isolated from the stems of *P. notoginseng* collected in September 2015 from Baoding, Hebei Province, P.R. China. The isolate was identified as *T. purpureogenus* by an analysis of the ITS region of the rDNA (GenBank Accession No. KY230505) and assigned the accession no. XL-025. A voucher specimen was deposited in School of Pharmaceutical Sciences, South-Central University for Nationalities. The fungus *T. purpureogenus* was inoculated aseptically into three 500 mL Erlenmeyer flasks each containing 300 mL of potato dextrose broth (PDB), and then cultured at 28 °C for 3 days with shaking at 160 rpm to afford the seed culture. The large-scale fermentation was performed into 150 flasks (500 mL), and each flask contained 80 g of rice and 80 mL glucose solution (20 g/L). Then, 5.0 mL of the seed culture was inoculated into each flask and incubated at room temperature for 50 days.

The harvested fermentation material was ultrasonically extracted three times with CHCl_3_/MeOH (1:1, v/v), and the organic solvent was evaporated under reduced pressure to yield a brown residue. The residue was then suspended in H_2_O and extracted three times with an equal volume of ethyl acetate (EtOAc) to yield 70 g of crude extract. The EtOAc extract was subjected to a silica gel column chromatography (CC) with a gradient mixture of CH_2_Cl_2_/MeOH (100:1–0:1) to afford eight fractions (Fr. A–Fr. H). Fraction C (1.2 g) was further purified by silica gel CC (petroleum ether/EtOAc = 1:15), Sephadex LH-20 (MeOH), and semi-preparative HPLC using with a solvent system of MeOH/H_2_O (96:4, 2 mL/min, 254 nm) to afford TalaA (25 mg, *t*_R_ = 20 min).

^1^H-NMR and ^13^C-NMR spectra were recorded on an Agilent DD2 (600 MHz) spectrometer in CD_3_OD using signals as internal standards (CD_3_OD, *δ*_H_ 3.30 ppm; *δ*_C_ 49.0 ppm). Silica gel (200–300 mesh, Anhui Liangchen Inc., China) and Sephadex LH-20 (Amersham Biosciences, Uppsala, Sweden) were employed for CC. Semi-preparative high-performance liquid chromatography (HPLC) was performed on a Lab Alliance instrument (Systems Inc., State College, Pennsylvania) using a Prevail C_18_ column (250 mm × 10 mm, 5 μm, GRACE Corporate, Columbia, MD, USA) and an UV detector (Mode 201). Finally the TalaA was evaporated into dry powder.

### Cell culture

CRC cell lines HCT116, SW480, and SW620 were obtained from the ATCC and cultured in DMEM (Hyclone, UT, USA) mixed with 10% FBS (Hyclone) and 1% penicillin/streptomycin antibiotics in 5% CO_2_ incubator at 37 °C. When the cells reached appropriate confluence, cells were treated with different concentrations of TalaA sulotion. The TalaA for cell treatment was dissolved into DMSO. The ferroptosis inhibitor ferrostatin-1 was purchased from MCE (HY-100579, MCE, China) and the ferroptosis inducer erastin was purchased from MCE (HY-15763, MCE, China). The ferroptosis suppressor protein 1 (FSP1) inhibitor iFSP1, GPX4 inhibitor FIN56, and iron chelating reagent—Deferiprone were all purchased from MCE, China.

### Cell activity measurement

Cell Counting Kit-8 (CCK8 for short) is a fast and highly sensitive test kit based on WST-8 that is widely used in the detection of cell activity. We used a commercial available CCK8 kit (C0037, Beyotime, China) to test the anti-cancer effect of TalaA against CRC cells including HCT116, SW480, and SW620 according to manufacturer’s instruction.

### Measurement of DNA synthesis rate by EdU method

To detect effects of compounds on cell proliferation is one of basic method to evaluate the antitumor activity of drug effect. It is widely accepted that the most accurate way is to directly detect the synthesis of DNA in cells. EdU (5-ethynyl-2′-deoxyuridine) is a thymidine analog which can be substituted for thymidine in DNA synthesis. We used the EdU-594 cell proliferation detection kit (C0078L, Beyotime, China) to examine the synthesis of DNA according to manufacturer’s instruction. The results of the EdU staining were photographed under a fluorescence microscope.

### Flow cytometry for cell cycle and apoptosis test

To examine the effects of the TalaA on the cell cycle, flow cytometry was employed. The cells were harvested by trypsin digestion after treatment, and fixed in 70% cold ethanol (in PBS) overnight at 4 °C. Then PI/RNase staining solution (#4087, cell signaling technology, USA) was added for 20 min (in dark) and the stained cells were tested and counted by flow cytometer. The cells for apoptosis analysis were harvested by trypsin treatment, and stained by Annexin V-FITC Early Apoptosis Detection Kit (#6592, Cell Signaling Technology, USA) for 20 min in darkness. The stained cells were counted and recorded by flow cytometer.

### Cell clone formation assay

Cell clone formation test is a powerful technique to detect cell proliferation ability or sensitivity to killing factors. Three hundred cells were cultured into 12-well plate. After incubation at 37 °C with 5% CO_2_ for 12 h, different concentrations of TalaA was added and the cells were cultured for 12 days (change media and TalaA every 3 days). After chemical treatment, the cells were fixed by 4% PFA for 10 min, and stained by crystal violet solution (C0121, Beyotime, China). The stained cells were photographed under a microscope.

### Cell membrane staining experiment

To study the effects of TalaA on cell membranes, we performed Dio-dye staining experiments. DiO, short for 3,3′-dioctadecyloxacarbocyanine perchlorate, is one of the most commonly used membrane of the fluorescent probe. DiO is a lipophilic membrane dye, which can gradually cause the whole cell membrane to be stained by lateral diffusion after entering the cell membrane. After cell culture and chemical treatment, the cells were washed by PBS and fixed by 4% PFA. Then the DiO cell membrane staining kit (C1038, Beyotime, China) was used to test the cell membrane according to the manufacturer’s instruction, and the stained cells were photographed under a fluorescence microscope.

### ROS test by H2DCFDA

H2DCFDA is a cell-permeable probe used to detect intracellular ROS. To detect the ROS induced by TalaA, the ROS-sensitive probe H2DCFDA (HY-D0940, MCE, China) was employed. After the cells were co-incubated with H2DCFDA staining solution, the fluorescent photographs were recorded via a fluorescence microscope. Moreover, the H2DCFDA-stained cells were also detected by flow cytometer.

### Cell microstructure test by transmission electron microscope

The SW480 cells with or without TalaA treatment were scraped by cell scraper and centrifuged at 400×*g* for 10 min. After the supernatant was discarded, 0.5% glutaraldehyde fixative solution was added into the tube to suspend the cells. After incubation at 4 °C for 10 min, the cells were centrifuged at 12,000×*g* for 10 min. Then, the supernatant was discarded, and 2.5% glutaraldehyde was slowly added along the wall to fix the cells. The photos of fixed cells were taken using transmission electron microscope (Hitachi HT7800, Japan) with different magnifications.

### Lipid peroxidation test

To test the lipid peroxidation, the cell-based lipid peroxidation assay kit (Abcam, USA) was used. This kit employed a sensitive ratiometric lipid peroxidation sensor which was able to change its fluorescence from red to green upon peroxidation by ROS in cells. CRC SW480 cells were stained with 1× lipid peroxidation sensor for 30 min at 37 °C. During the last 10 min of incubation, hoechst 33342 was added to satin the cell nucleus. After incubation, cells were washed three times with HHBS and imaged with a fluorescence microscope.

### RNA sequencing

To reveal transcriptome changes caused by TalaA, the RNA was extracted by TRIzol reagent (ThermoFisher Scientific, USA) from SW480 cells with or without TalaA treatment. The library construction and RNA-sequencing were completed by Novogene Company (Novogene, Beijing, China).

### Reverse transcriptional PCR and real-time PCR

The RNA was extracted by TRIzol reagent (ThermoFisher Scientific, USA) from SW480 cells with different concentrations of TalaA treatment. The cDNA was synthesized with a cDNA synthesis kit (D7170M, Beyotime, China), and the real-time PCR was performed using a SYBR Green qPCR kit (D7260, Beyotime, China) in a light cycler 480 II (Roche, USA). The primer sequences for real-time PCR are listed in Table 1.

**Table 1:** The primer list for real-time PCR.

Name	q-PCR primer
FTL	Forward: 5′-GCTCCCAGATTCGTCAGAA-3′ Reverse: 5′-CCCAGAGAGAGGTAGATGTAGG-3′
FTH1P23	Forward: 5′-CAGGTGAAAGCCATCAAAGA-3′ Reverse: 5′-TTATCACTGTCTCCCAGCG-3′
SAT2	Forward: 5′-TGATTCGGGTGAAGACTGC-3′ Reverse: 5′-TCTCCAAAGCCATCTGCTC-3′
HMOX1	Forward: 5′-ATTGCCAGTGCCACCAAGT-3′ Reverse: 5′-TGAGCAGGAACGCAGTCTT-3′
ALOXE3	Forward: 5′-TGTATTTCGCTTTCCTGACC-3′ Reverse: 5′-CTTGTTTGCTTGCCTCTGA-3′
ACSL5	Forward: 5′-TAGAAGCACTGAGAGATGCG-3′ Reverse: 5′-TTGTGAACAGCAGCAGGA-3′
SLC7A11	Forward: 5′-TATCCCTGGCATTTGGAC-3′ Reverse: 5′-TCACTACAGTTATGCCCACAG-3′
GSS	Forward: 5′-ACAGGATGACTTTACCGCTC-3′ Reverse: 5′-AGCGGTAAAGTCATCCTGTT-3′

### Western blotting

To detect the protein level alteration of ferrotosis-related molecules, western blotting was performed. The cell samples were lysed by RIPA buffer containing 0.1% SDS. After running the SDS–PAGE, the protein samples were transferred onto the PVDF membrane, and followed by antibody incubation. The antibodies were listed as follows: rabbit anti-SLC7A11 (1:1000, PA1-16893, Invitrogen, USA), rabbit anti-ALXOE3 (1:800, ab118470, Abcam, USA), and mouse anti-β-actin (1:5000, A5441-100UL, Sigma, USA). The blots were explored with ECL, and the immunoreactive signals were generated in a luminescence detection system using horseradish peroxidase-labeled secondary antibody.

### Gene overexpression

The SLC7A11 overexpression plasmid pRP[Exp]-EF1A-hSLC7A11 was constructed in VectorBuilder company (Guangzhou, China). The SLC7A11 overexpression plasmid was transfected into SW480 cells using lipofectamine 3000 (L3000-015, Life Technologies, CA, USA) according to the manufacturer’s instruction.

### Gene knockdown with shRNA

ShRNA interference fragments were designed for human SLC7A11 and ALOXE3. These interference fragments were constructed into the downstream U6 promoter of the *Age*I-digested and *EcoR*I-digested lentivirus vector (pLKD-CMV-EGFP-2A-Puro-U6-shRNA) by molecular biological means. The shRNA fragment sequences were shown as following:

SLC7A11 shRNA1:

Top Strand: 5′-CCGGGGGAGTCTCCATTATCATTGGTTCAAGAGACCAATGATAATGGAGACTCCCTTTTTTG-3′

Bottom Strand: 5′-AATTCAAAAAAGGGAGTCTCCATTATCATTGGTCTCTTGAACCAATGATAATGGAGACTCCC-3′

SLC7A11 shRNA2:

Top Strand: 5′-CCGGGCAGCTACTGCTGTGATATCCTTCAAGAGAGGATATCACAGCAGTAGCTGCTTTTTTG-3′

Bottom Strand: 5′-AATTCAAAAAAGCAGCTACTGCTGTGATATCCTCTCTTGAAGGATATCACAGCAGTAGCTGC-3′

ALOXE3 shRNA1:

Top Strand: 5′-CCGGGCCTTGACAAAGACGACAACTTTCAAGAGAAGTTGTCGTCTTTGTCAAGGCTTTTTTG-3′

Bottom Strand: 5′-AATTCAAAAAAGCCTTGACAAAGACGACAACTTCTCTTGAAAGTTGTCGTCTTTGTCAAGGC-3′

ALOXE3 shRNA2:

Top Strand: 5′-CCGGGGAAGAAGCTGGATGACATGCTTCAAGAGAGCATGTCATCCAGCTTCTTCCTTTTTTG-3′

Bottom Strand: 5′-AATTCAAAAAAGGAAGAAGCTGGATGACATGCTCTCTTGAAGCATGTCATCCAGCTTCTTCC-3′

### Glutathione examination

After being washed by PBS, cells were collected by cell scraper into a 1.5 ml EP tube. Super sonication was performed to lyse the cells. After centrifuge, the glutathione in supernatant was detected by a glutathione assay kit (S0053, Beyotime, China) following the manufacturer’s instruction.

### Xenograft

5 × 10^6^ HCT116 cells were inoculated subcutaneously in the underarm of Balb/c nude female mice (5-week old). The inoculated mice were randomly divided into two groups (6 mice each group). When the tumor reached 300 mm^3^, the drug group was given TalaA intraperitoneally at a dose of 6.0 mg/kg, and the control group was given the same dose of cosolvent—corn oil. The drug (or cosolvent) was injected every 2 days. Body weight and tumor volume were measured every 2 days. After the mice were sacrificed, the tumor was taken and fixed with 10% formalin. The animal experiments are carried out in accordance with animal ethics. All protocols and procedures were approved by the Institutional Review Committee of Jining Medical University for animal warfare.

### IHC staining

The formalin-fixed tumors were embedded into paraffin blocks, and the blocks were dissected into 4.0 μM thickness slices. In IHC staining experiment, citric acid buffer boiling method is used to unmask and repair the antigen on the slides. The primary antibodies used in IHC were listed as following: Ki67 (ab15580, Abcam, USA) was diluted 1:400 with 1% BSA in PBS, SLC7A11 (26864-1-AP, Proteintech, China) was diluted 1:200 with 1% BSA in PBS, HMOX1 (10701-1-AP, Proteintech, China) was diluted 1:200 with 1% BSA in PBS.

### Statistical analysis

When comparing the two groups of data, SPSS was used to analyze whether the data were normally distributed. For the comparison between two groups of data with normal distribution, the method of *t*-test was employed. When *p* value < 0.05, the differences were considered as significant.

## Results

### Purification and identification of TalaA

TalaA was purified from the solid fermentation cultures of an endophytic fungus *T. purpureogenus* isolated from the stems of *P. notoginseng*, and its purity was detected by HPLC (as shown in Fig. S1). As shown in Fig. S2, the chemical structure of TalaA was characterized by comparison of its NMR data with literature value^26^: ^1^H-NMR (600 MHz, CD_3_OD) *δ*_H_: 7.73 (1H, s, H-4), 7.49 (2H, d, *J* = 8.6 Hz, H-8 and H-12), 6.86 (2H, d, *J* = 8.6 Hz, H-9 and H-11), 6.47 (1H, s, H-6), 5.38 (1H, s, H-17), 4.72 (1H, d, *J* = 9.5 Hz, H-25), 3.95 (1H, dd, *J* = 7.7, 12.3 Hz, H-14), 3.22 (1H, d, *J* = 7.6 Hz, H-15), 2.12 (1H, m, H-26), 1.84 (1H, t, *J* = 12.0 Hz, H-22), 1.71 (1H, m, H-19), 1.70 (1H, m, H-20a), 1.69 (1H, m, H-21a), 1.50 (3H, s, H_3_-31), 1.48 (1H, m, H-18a), 1.45 (3H, s, H_3_-32), 1.24 (1H, m, H-27a), 1.03 (1H, m, H-21b), 1.02 (1H, m, H-27b), 0.95 (3H, s, H_3_-30), 0.94 (1H, m, H-20b), 0.93 (1H, m, H-18b), 0.86 (3H, d, *J* = 6.1 Hz, H_3_-29), 0.76 (3H, t, *J* = 7.2 Hz, H_3_-28), 0.69 (3H, d, *J* = 6.6 Hz, H_3_-33); ^13^C NMR (150 MHz, CD_3_OD) *δ*_C_: 198.3 (s, C-13), 171.8 (s, C-2), 160.7 (s, C-10), 146.9 (d, C-4), 137.8 (d, C-25), 137.5 (d, C-17), 134.9 (s, C-24), 134.0 (s, C-5), 133.4 (d, C-8 and C-12), 131.6 (s, C-3), 131.3 (s, C-16), 127.1 (s, C-7), 123.7 (d, C-6), 117.3 (d, C-9 and C-11), 52.4 (d, C-15), 50.7 (d, C-14), 49.9 (t, C-18), 41.4 (d, C-22), 37.1 (t, C-20), 36.3 (s, C-23), 35.3 (d, C-26), 31.5 (t, C-27), 28.7 (d, C-19), 25.4 (t, C-21), 23.3 (q, C-29), 22.6 (q, C-31), 21.1 (q, C-33), 21.0 (q, C-30), 14.4 (q, C-32), and 12.5 (q, C-28). The NMR identified TalaA structural formula with atomic number as shown in Fig. S3.

### TalaA-suppressed CRC proliferation

**Fig. 1:**
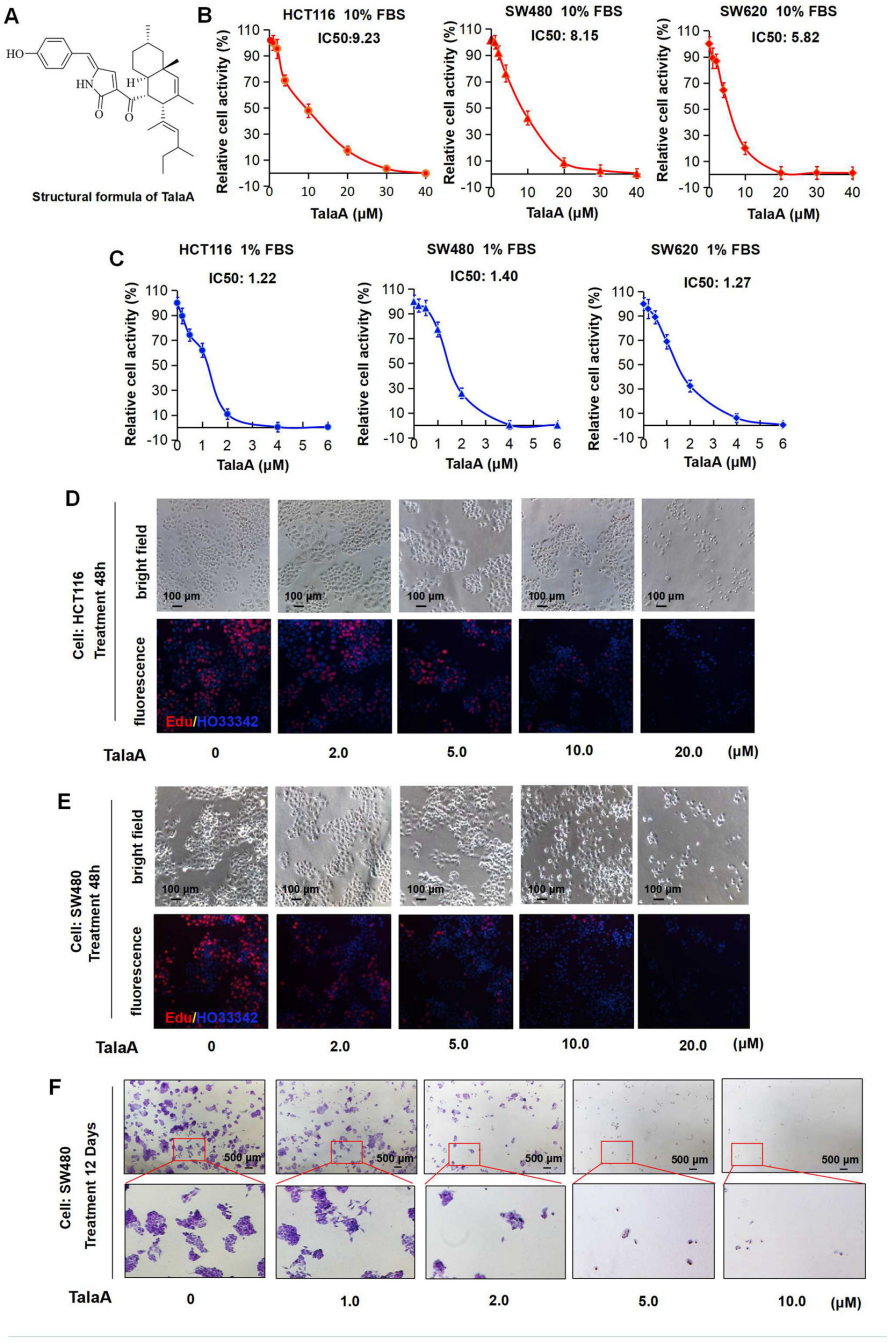
TalaA killed colorectal cancer cells. **A** The structure of TalaA. **B** CRC cells were incubated with TalaA in DMEM media containing 10% FBS for 24 h, then the CCK8 kit was employed to examine the cells activities. From left to right, the cells were HCT116, SW480, and SW620, respectively. For each concentration point, three repeats were performed. **C** CRC cells were incubated with TalaA in DMEM media containing 1% FBS for 24 h, then the CCK8 kit was employed to examine the cells activities. From left to right, the cells were HCT116, SW480, and SW620, respectively. For each concentration point, three repeats were performed. **D** After HCT116 cells were incubated with TalaA in 10% FBS contained media for 48 h, Edu solution was added and cells were stained according to manufacturer’s instruction. Red spots meant Edu-positive cells, and blue spots meant Hoechst33342-positive cells. **E** After SW480 cells were incubated with TalaA in 10% FBS contained media for 48 h, Edu solution was added and cells were stained according to manufacturer’s instruction. Red spots meant Edu-positive cells, and blue spots meant Hoechst33342-positive cells. **F** The crystal violet staining results for the clonogenicity of SW480 cells. The SW480 cells were cultured with 0–10 μM TalaA for 12 days.

TalaA suppressed the cell growth of CRC cell lines HCT116, SW480, and SW620 in dose-dependent (Fig. 1B, C) and time-dependent manners (Fig. S4). As can be seen in Fig. 1B, in high-concentration (10%) FBS medium, the IC_50_ values of TalaA in HCT116, SW480, and SW620 cells were 9.23, 8.15, and 5.82 μM, respectively. In the low-concentration (1%) FBS medium, the IC_50_ values of TalaA in HCT116, SW480, and SW620 cells were 1.22, 1.40, and 1.27 μM, correspondingly. Besides, we measured the cell activity via determination of the rate of DNA synthesis. Our EdU experiments showed that the treatment with TalaA decreased DNA synthesis (Fig. 1D, E). Furthermore, the results of the clone formation experiments also clearly revealed the anticancer function of TalaA against CRC cells (Fig. 1F). Morphologically, the cells treated with TalaA lost their original natural morphological characteristics, indicating that TalaA not only suppressed cell growth but also induced cell death.

### TalaA killed CRC cells via ROS level elevation but not apoptosis

**Fig. 2:**
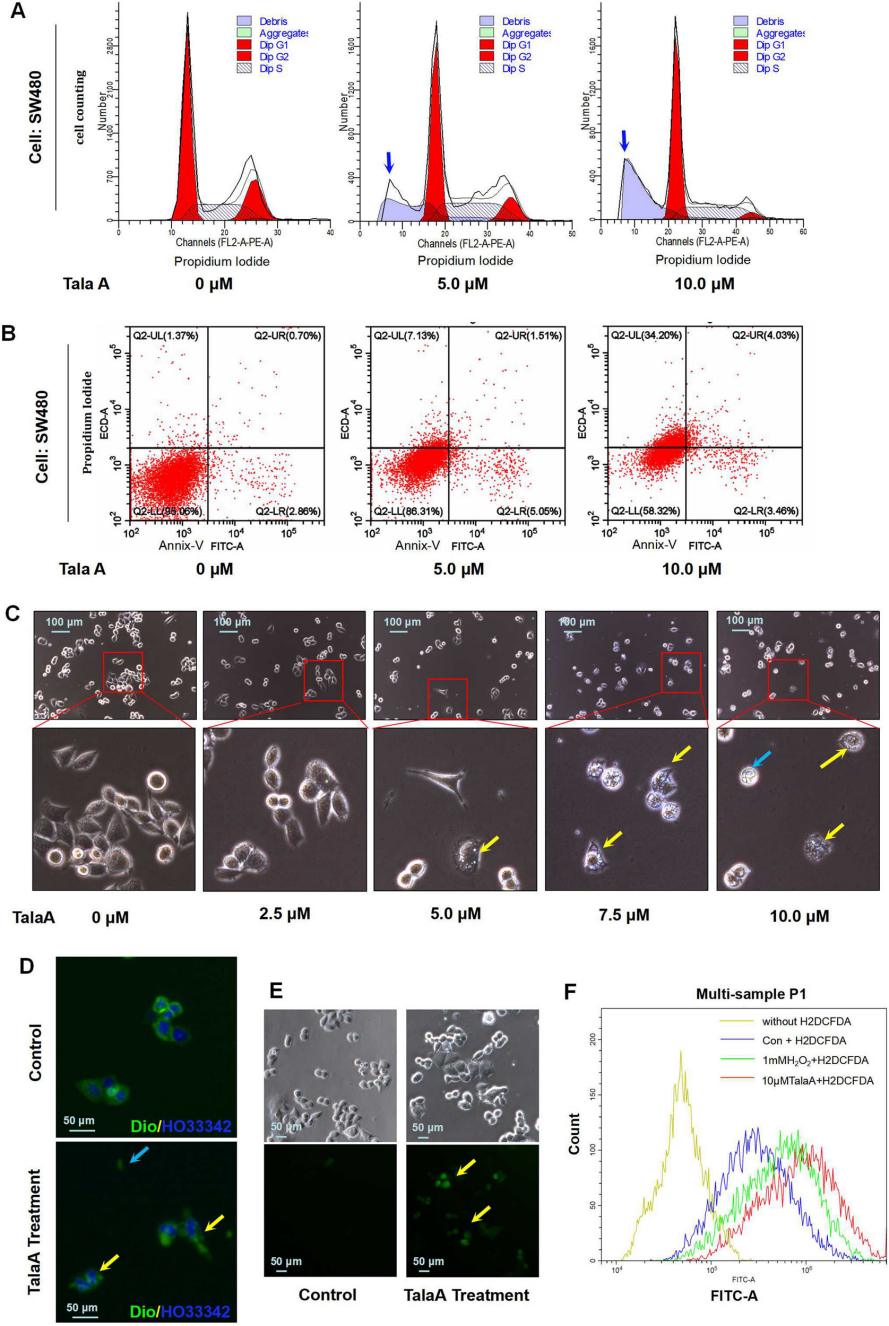
TalaA elevated ROS in CRC cells. **A** The SW480 cells were co-incubated with or without TalaA for 24 h, and the cells were stained by PI/RNase. And the stained cells were detected by flow cytometry to examine the cell cycle. The two red peaks represent G1 and G2 stage, and the cross-court area represents S stage. The blue arrow means the dead cell debris. **B** The SW480 cells were co-incubated with or without TalaA for 24 h, and the cells were stained by PI and Annexin V-FITC. The stained cells were detected by flow cytometry to examine the apoptosis. **C** The SW480 cells were treated by 0–10 μM TalaA for 24 h, and the cellular morphology was recorded by microscope. Note: cells with membranes perforated were marked with yellow arrows; and the dead cells were marked with blue arrows. **D** The SW480 cells were treated by 8.0 μM for 24 h, and the cell membrane and nucleus were stained by DiO (green) and Hoechst 33342 (blue). Note: the yellow arrow indicated damaged membrane and the blue indicated membrane fragment without nucleus. **E** The SW480 cells were treated by 8.0 μM for 4 h, and the ROS was detected by H2DCFDA. The yellow arrow indicated ROS increased cells. **F** The SW480 cells treated with TalaA or H_2_O_2_ were incubated with 5 µM H2DCFDA in PBS in the dark for 30 min at 37 °C. After being digested, the H2DCFDA-stained cells were detected by flow cytometer.

To investigate the mechanism of TalaA-induced CRC cell growth inhibition, we examined its effects on the cell cycle and apoptosis of CRC cells. As illustrated in Fig. 2A, TalaA did not cause cell cycle arrest, but increased the subG1 peak, indicating that it can induce cell death. Moreover, TalaA did not significantly induce early apoptosis (Fig. 2B), but triggered other programmed death mechanisms in CRC cells. Interestingly, TalaA treatment led to drastic changes in the morphology of the cell membrane: the membrane surface was no longer smooth and intact, but had multiple perforations (Fig. 2C). To determine the integrity of the cell membrane, we used a membrane/nucleus double-staining kit. Notably, TalaA caused a loss of the original integrity of the cell membrane (Fig. 2D). The ability of TalaA to change cell morphology, destroy membrane integrity, and elevate ROS levels without induction of apoptosis indicated that TalaA might trigger a novel cell death pathway other than apoptosis. Through H2CDFDA staining, we found that TalaA treatment obviously increased ROS levels in CRC cells (Fig. 2E), which was also supported by the results from flow cytometry (Fig. 2F).

### TalaA-induced ferroptosis in CRC cells

**Fig. 3:**
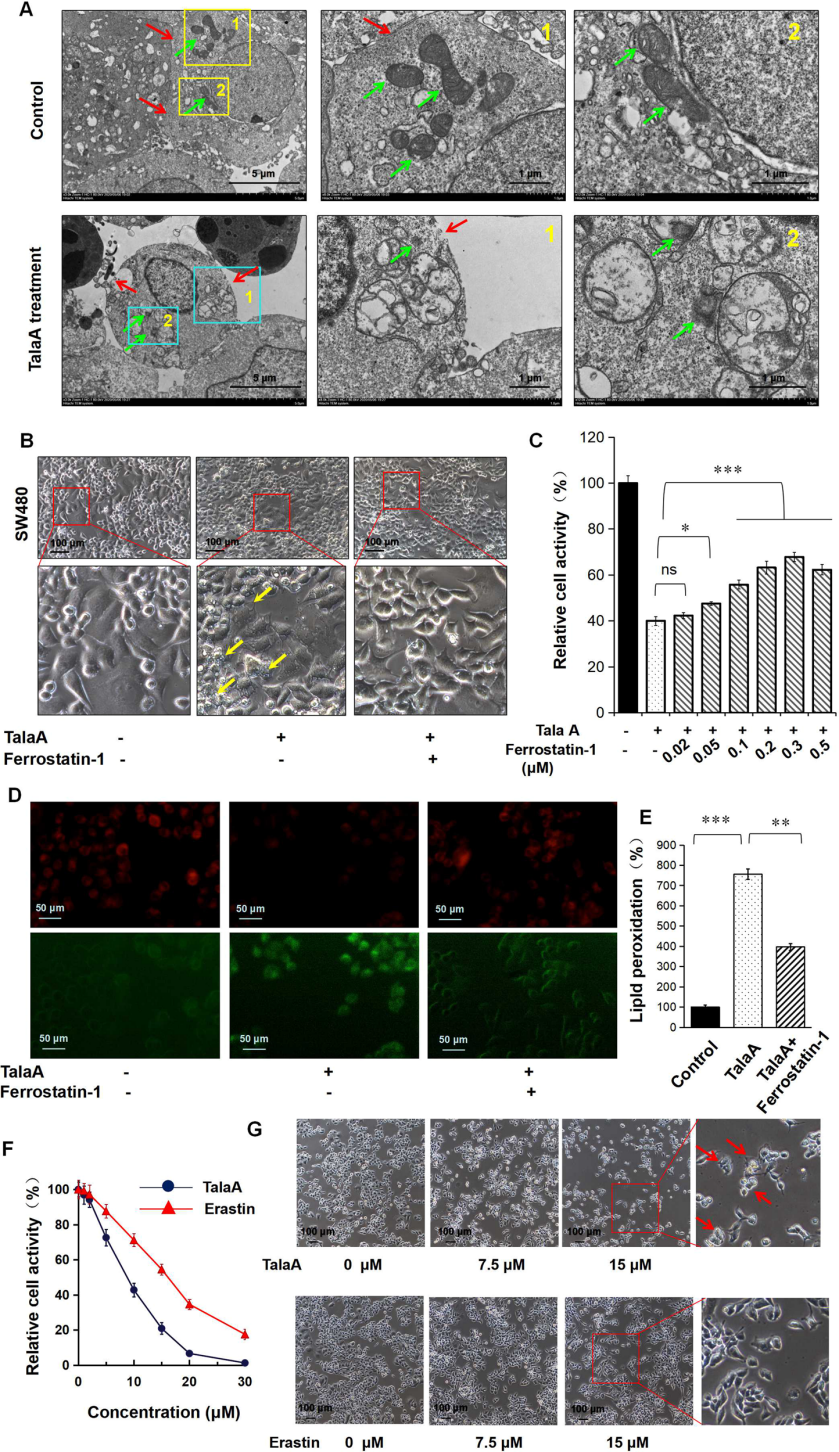
TalaA-induced ferroptosis in CRC cells. **A** Transmission electron microscopy is used to observe the microscopic substructure of cells: The SW480 cells were treated by 5.0 μM TalaA for 24 h and fixed by 2.5% glutaraldehyde. The fixed cells were taken photos using transmission electron microscope (Hitachi HT7800, Japan) with different magnifications (the magnification was shown in picture). The green arrow indicates mitochondria and red arrow indicates membrane. After being treated by TalaA, the mitochondria are wrinkled, with the internal crest disappearing; and the cell membrane broken. **B** The SW480 cells pretreated by 0.1 μM ferrostatin-1 were co-incubated with 10.0 μM TalaA for 12 h. Then the cellular morphology was recorded by microscope. (The cells that look like membranes perforated were marked with yellow arrows.) **C** The SW480 cells pretreated by 0–0.5 μM ferrostatin-1 were co-incubated with 10 μM TalaA for 24 h, and the cells were tested by CCK8 kit. Error bars means SD, *N* = 3 independent repeats. *p* values were calculated using two-tailed unpaired Student’s *t*-test, * means *p* < 0.05; *** means *p* < 0.001 versus TalaA treatment. **D** The lipid peroxidation was detected by cell-based lipid peroxidation assay kit. The lipid peroxidation sensor changes its fluorescence from red to green upon peroxidation by ROS in cells. The stained cells were taken photos in a fluorescence microscope. **E** The ratio of green fluorescence to red fluorescence was calculated with Image J software to show the degree of lipid peroxidation. **F** Comparison of the anti-cancer effect between erastin and TalaA on colon cancer cells. Colorectal cancer SW480 cells were treated by different concentrations of TalaA and erastin respectively, for 24 h, and the relative cell activity was detected with CCK8 kit. Red points represent TalaA treatment group, and blue triangles represent erastin treatment group. For each concentration point, three repeats were performed. **G** SW480 cells were co-incubated with 0, 7.5, 15 μM TalaA and erastin, respectively. After 48 h, the cultured cells were taken pictures with phase contrast microscope. To compare the morphological alteration, the photos of 15 μM TalaA and erastin-treated SW480 cells were amplified. The red arrows indicated dead cells with obvious morphological alteration.

As can be seen in Fig. 3A, TalaA-treated cells showed the typical subcellular morphological characteristics of ferroptosis: cell membrane vesicles or ruptures, smaller or shriveled mitochondria, and decreased or disappeared mitochondrial cristae. All aforementioned morphological features characterize ferroptosis. We pretreated the CRC cells with ferrostatin-1, a strong inhibitor of ferroptosis, and then performed cell treatment with TalaA. It is noteworthy that ferrostatin-1 significantly alleviated TalaA-caused membrane perforations (Fig. 3B). Moreover, ferrostatin-1 dose-dependently neutralized TalaA-induced cell death (Fig. 3C), which indicated that ferroptosis is the critical mechanism by which TalaA kills CRC cells. To verify this hypothesis, transmission electron microscopy was employed to observe the cell membrane and mitochondria, which could vividly reflect the subcellular morphological characteristics of ferroptosis. Besides, we also confirmed that TalaA induced the death of colon cancer cells by the ferroptosis pathway. As depicted in Fig. S5, deferiprone, an iron-chelating agent, partially alleviated the cell death caused by TalaA in a dose-dependent manner. Furthermore, we found that TalaA increased lipid peroxidation (Fig. 3D, E), which is an essential step and landmark of ferroptosis and deferiprone neutralizd TalaA-induced lipid peroxidation. Impressively, TalaA killed CRC cells more effectively than erastin, a known ferroptosis inducer (Fig. 3F). The differences between the anticancer effects of TalaA and erastin from morphological perspective can be seen in Fig. 3G.

### TalaA-induced ferroptosis was verified by RNA-seq analysis

**Fig. 4:**
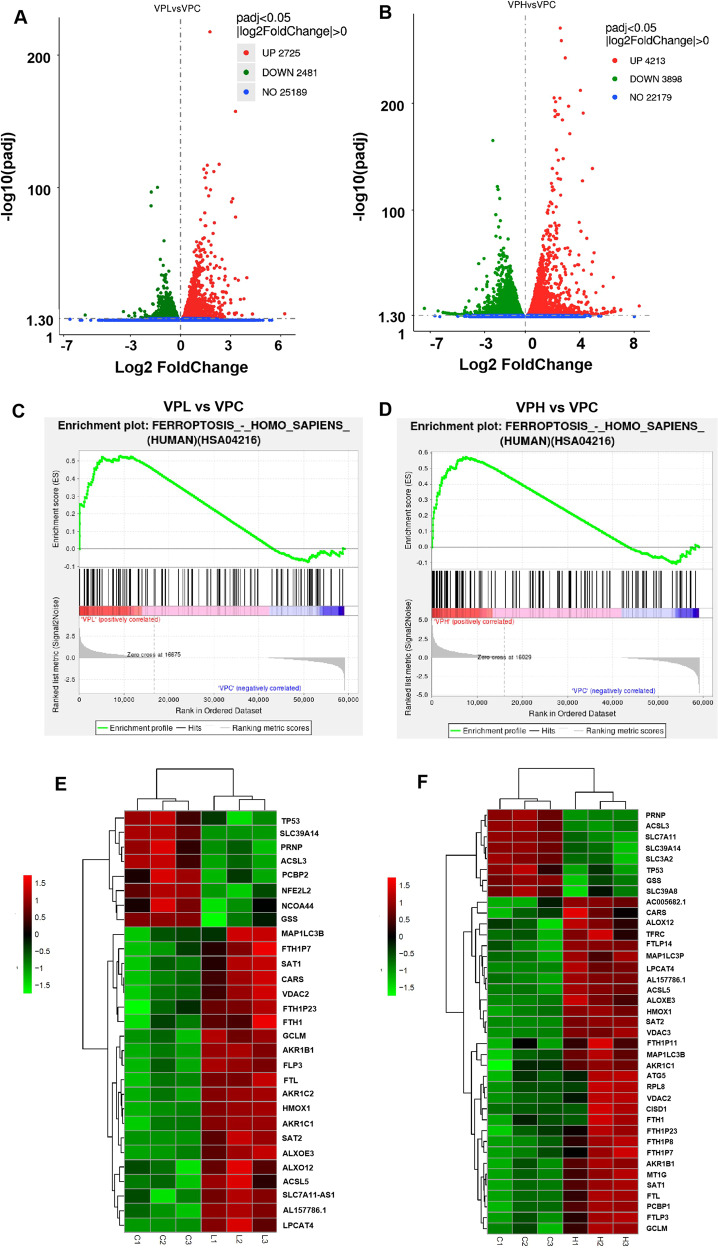
TalaA treatment-induced transcriptome alteration. The SW480 cells were treated with two concentrations of TalaA for 12 h. **A** Volcano plot showing the differences in RNA expression after 5.0 μM TalaA treatment. VPL means low concentration (5.0 μM) TalaA treatment; VPC means no chemical treatment (DMSO Control). **B** The volcano plot showing the differences in RNA expression after 10.0 μM TalaA treatment. VPH means high concentration (10.0 μM) TalaA treatment; VPC means no chemical treatment (DMSO control). **C** Through KEGG-enrichment analysis, it was found that the ferroptosis pathway molecules were up-regulated by 5.0 μM TalaA. **D** KEGG-enrichment analysis showed that the ferroptosis pathway molecules were up-regulated by 10.0 μM TalaA. **E** The heatmap data showed 5.0 μM TalaA resulted in gene expression alteration of ferroptosis-correlated molecules including FTL, SAT2, ALOXE3, GSS, ALOX12, HMOX1, ACSL5, and so on. **F** The heatmap data showed 10.0 μM TalaA resulted in gene expression alteration of ferroptosis-correlated molecules including FTL, SLC7A11, HMOX1, ALOXE3, SAT1, SAT2, GSS, ACSL5, ALXO12, PCBP1, MAP1LC3P, and so on.

Transcriptome sequencing was performed to further investigate the mechanism by which TalaA causes CRC cell death. Figure 4A displays the changes in the transcriptome induced by a low concentration (5.0 μM) of TalaA, while Fig. 4B depicts the alterations in the transcriptome caused by a high concentration (10.0 μM) of TalaA. The low TalaA concentration led to upregulation of 2725 transcripts and downregulation of 2481 transcripts. On the other hand, the high concentration of TalaA induced the upregulation of 4213 transcripts and the downregulation of 3898 transcripts. Then, we conducted functional enrichment analysis of differentially expressed genes. Remarkably, the KEGG analysis results were consistent with our speculation that TalaA could induce ferroptosis. We based our conclusion on the following observations: (1) ferroptosis pathway was among the top pathways established by KEGG analysis in the treatments with both the low and high concentrations of TalaA (Fig. 4C, D); (2) the constructed heat map showed that the low concentrations of TalaA led to significant changes in 29 genes closely related to ferroptosis, whereas the high concentrations of the compound caused significant changes in 39 genes tightly associated with ferroptosis (Fig. 4E, F). Additionally, the levels of most of the ferroptosis-related molecules were altered by TalaA in a concentration-dependent manner. The RNA-Seq results were uploaded and are publicly available in the Sequence Read Archive (SRA) database (https://www.ncbi.nlm.nih.gov/sra) under accession number PRJNA637941. Using two independent approaches, we clearly evidenced that TalaA induces ferroptosis (Figs. 3 and 4).

### RT-qPCR verification

**Fig. 5:**
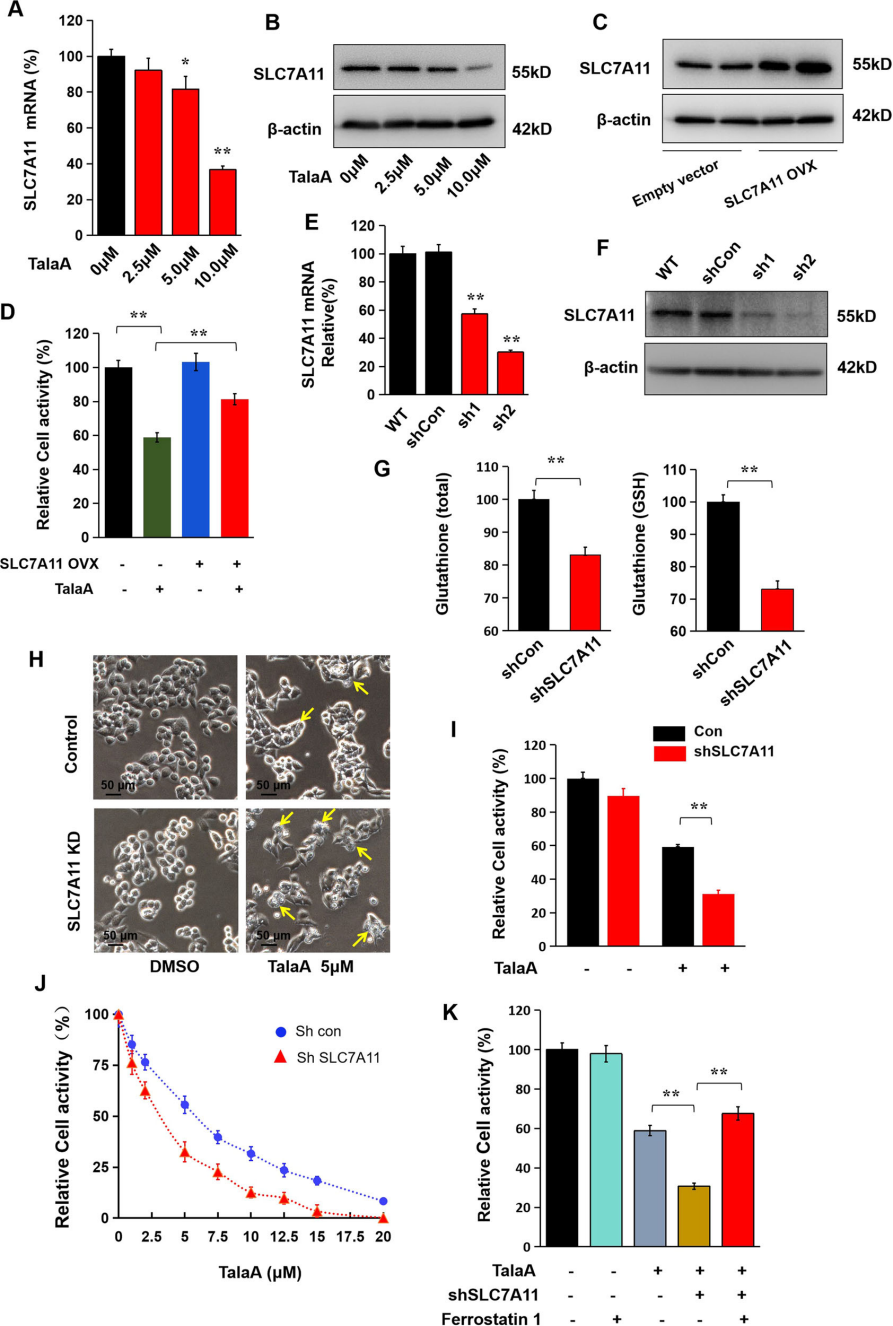
TalaA accelerated ferroptosis in CRC cells by down-regulation of SLC7A11. **A** SLC7A11 mRNA was decreased by TalaA dose-dependently; *p < 0.05, **p < 0.01, *N* = 3 independent repeats. **B** SLC7A11 protein level was decreased by TalaA in a dose-dependent manner. **C** The SLC7A11 protein level was increased by SLC7A11 overexpression plasmid (SLC7A11 OVX) transfection. **D** The relative cell activities of SLC7A11-overexpressed cells and control cells after treated by 5.0 μM TalaA. **p < 0.01, *N* = 3 independent repeats. **E** The mRNA expression was suppressed by SLC7A11-specific lenti-shRNA; **p < 0.01 versus shCon, *N* = 3 independent repeats. **F** The SLC7A11 protein level was decreased by lenti-shSLC7A11. **G** Total glutathione and reduced glutathione was decreased as SLC7A11 being knocked down; **p < 0.01, *N* = 3 independent repeats. **H** 5.0 μM TalaA induced slight cell membrane to get destroyed in wild type SW480 cells. However same concentration of TalaA induced strong membrane to get destroyed in SLC7A11 knocked down SW480 cells. The yellow arrows indicate membrane-damaged cells. **I** 5.0 μM TalaA-treated SLC7A11 knocked-down SW480 cells had lower cell activity than wild type SW480 with same concentration TalaA treatment; **p < 0.01, *N* = 3 independent repeats. **J** The scatter plot of TalaA inhibited cell growth. The blue points represented wild type SW480, and red squares represented SLC7A11 knocked down SW480 cells. For each concentration point, three repeats were performed. **K** The SLC7A11 knockdown SW480 and wild type SW480 were treated by 5.0 μM TalaA with or without Ferrostatin-1. The cell activity of SW480 cells was detected with CCK8 kit; **p < 0.01, *N* = 3 independent repeats.

**Fig. 6:**
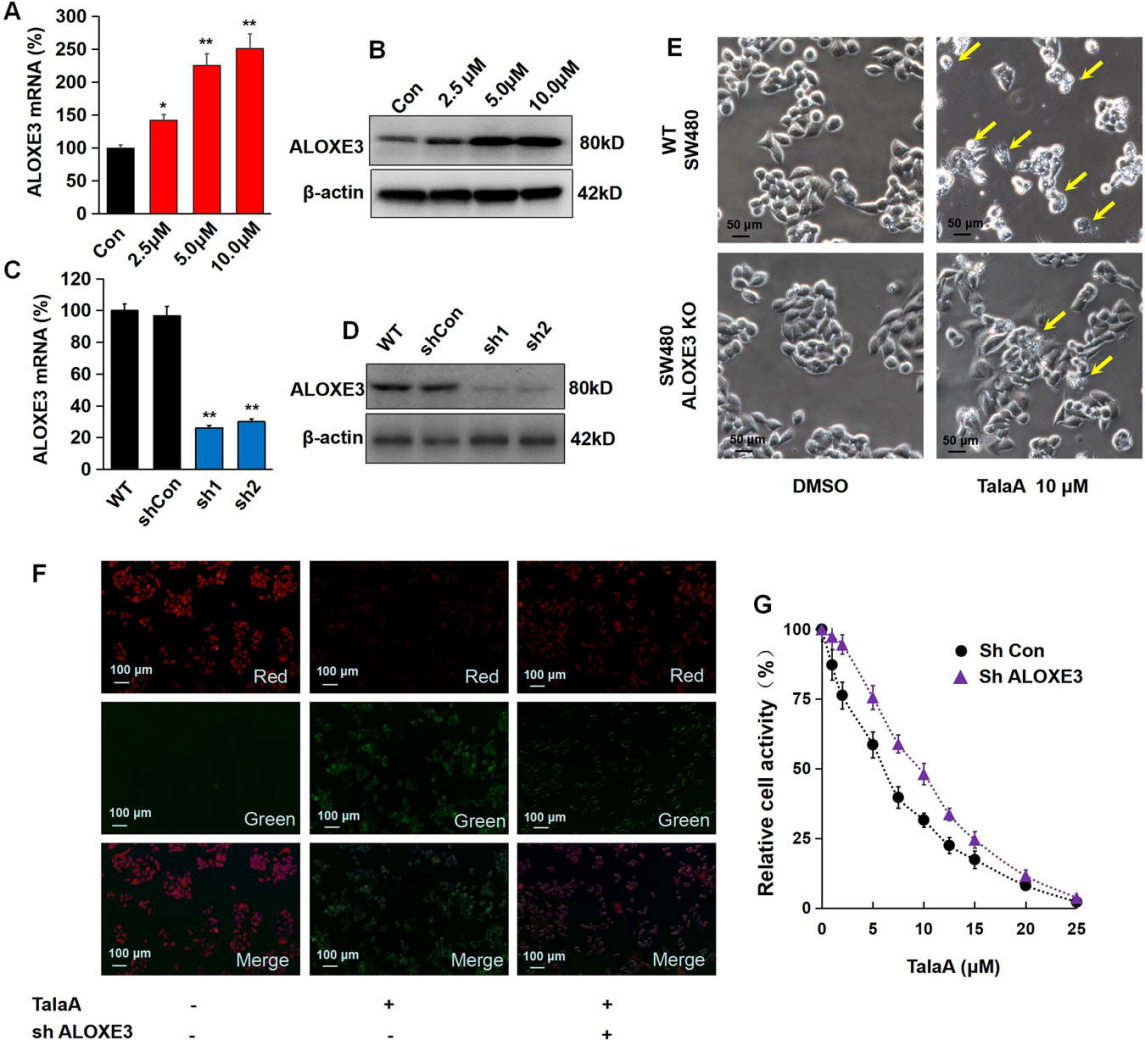
TalaA enhanced ferroptosis in CRC cells by up-regulation of ALOXE3. **A** ALOXE3 mRNA was increased by TalaA dose-dependently; *p < 0.05, **p < 0.01, *N* = 3 independent repeats. **B** The protein level of ALOXE3 was elevated by TalaA in a dose-dependent manner. **C** The mRNA level was decreased via lenti-shALOXE3 infection. **p < 0.01 versus ShCon, *N* = 3 independent repeats. **D** The ALOXE3 protein level was reduced by lenti-shALOXE3. **E** Although 10 μM TalaA violently caused cell membrane destroy in wild type SW480 cells, same concentration TalaA only led to mild membrane destroy in ALOXE3 knocked down SW480 cells. The yellow arrows indicated broken cells. **F** The lipid peroxidation was detected by cell-based lipid peroxidation assay kit. The stained cells were recorded with a fluorescence microscope. When the lipids were peroxidized, the fluorescence shifted from red to green. **G** The cell activity curve right shifted as ALOXE3 was knocked down. The black points represented wild type SW480, and purple triangles represented ALOXE3 knocked down SW480 cells. For each concentration point, three repeats were performed.

To further verify our hypothesis and confirm the above transcriptome-sequencing results, we assessed the expression levels of eight important ferroptosis-related molecules by reverse transcription-PCR, followed by real-time qPCR. As can be seen in Figs. 5A, 6A, and S6, ferroptosis positively correlated molecules such as FTL, FTH1P23, SAT2, HMOX1, ALOXE3, and ACSL5 were upregulated by TalaA. Conversely, the ferroptosis negatively correlated molecules such as SLC7A11 and SLC39A14 were downregulated by TalaA.

### TalaA-induced ferroptosis by SLC7A11 downregulation

We found that TalaA dose-dependently decreased the expression of SLC7A11 in SW480 cells (Fig. 5A, B). To investigate the involvement of SLC7A11 in TalaA-caused ferroptosis, we overexpressed SLC7A11 in SW480 cells (Fig. 5C). As can be observed in Fig. 5D, the cell activity of TalaA-treated SLC7A11-overexpressed cells was significantly higher than that of TalaA-treated control cells. This result indicated that SLC7A11 might play an important role in TalaA-induced cell death. To further confirm the role of SLC7A11 in TalaA-induced ferroptosis, we knocked down *SLC7A11* (gene for a channel protein important for cystine transmembrane transport) in SW480 cells (Fig. 5E, F). Additionally, cystine is an essential component in the synthesis of glutathione, which is a crucial antioxidant in cells. The knockdown of *SLC7A11* decreased the glutathione level (Fig. 5G) and increased the sensitivity to TalaA (Fig. 5H–J). As seen in Fig. 5K, the ferroptosis inhibitor ferrostatin-1 neutralized the anticancer effects caused by *SLC7A11* knockdown and TalaA treatment. It is obvious from Fig. 5 (entire panel) that SLC7A11 had an essential part in cell resistance to ferroptosis; TalaA decreased the SLC7A11 level, thereby aggravating ferroptosis. Besides, because of the important role of GPX4 in ferroptosis, we investigated the combined effect of TalaA and GPX4 inhibitor-FIN56 on SW480 cells. The GPX4 inhibitor-FIN56 enhanced the anticancer effect of TalaA, showing that TalaA might induce ferroptosis through pathways other than GPX4 (Fig. S7).

### TalaA-induced ferroptosis by ALOXE3 upregulation

Figure 6A displays the dose-dependent increase in the level of ALOXE3 mRNA by TalaA, which was consistent with the obtained RNA-Seq results. Western blotting data confirmed that TalaA elevated the protein level of ALOXE3 in SW480 cells (Fig. 6B). To investigate the role of ALOXE3 in TalaA-induced ferroptosis, we knocked down *ALOXE3* in SW480 cells (Fig. 6C, D), which revealed a lower degree of destruction caused by TalaA treatment than that of the wild-type cells (Fig. 6E). That is to say, the TalaA-induced perforation of the cell membrane was alleviated by *ALOXE3* knockdown. Furthermore, during the knock-down of *ALOXE3*, the extent of lipid peroxidation caused by the same concentration of TalaA was obviously reduced (Fig. 6F). Then, the concentration-dependence curve of the TalaA-inhibited CRC cell growth was analyzed. It is visible from Fig. 6G that the quantification curve of TalaA-caused cell growth inhibition shifted to the right, because of *ALOXE3* knockdown, which showed the importance of *ALOXE3* as a ferroptosis accelerator. TalaA triggered ferroptosis by upregulation of ALOXE3, which increases lipid peroxidation, the critical trigger for ferroptosis.

### TalaA suppressed xenograft tumor growth in vivo

**Fig. 7:**
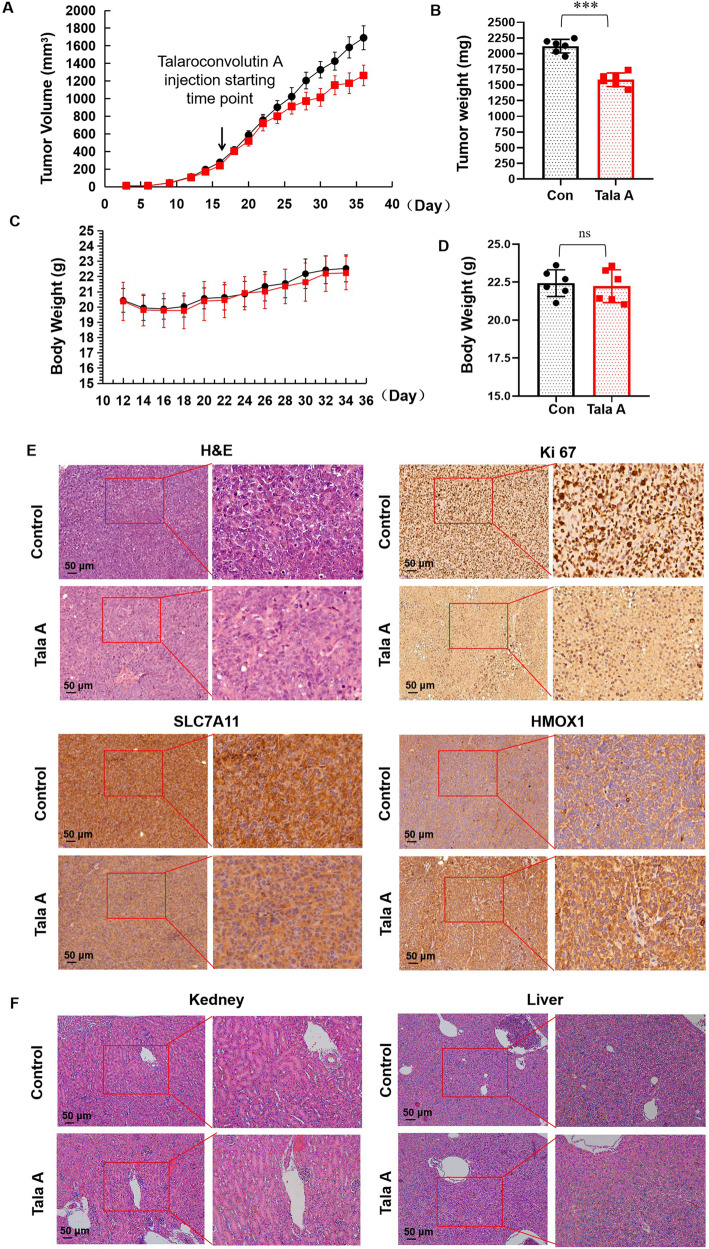
TalaA inhibited xenografted tumor growth in vivo. **A** Tumor column was recorded. The black points represented blank control group (corn oil), and the red squares TalaA treatment group (six mice for each group). **B** The final tumor weight was compared between the two groups: ***p < 0.001 indicated the significant difference. **C** Mice body weight was recorded. The black points represented blank control group and red squares TalaA treatment group. **D** The final body weight was compared between the two groups: no significant difference between the two groups; “ns” represent no significant difference. **E** Pathological staining for xenografted tumors of the above two groups: H&E staining photos and IHC staining for Ki67, SLC7A11, and HMOX1 photos for both control group and TalaA treatment group. **F** The mice liver and kidney were fixed in the formalin and stained with H&E dye for both control group and TalaA treatment group.

To detect the antitumor effect of TalaA, Balb/c nude mice were inoculated with cancer HCT116 cells. Next, mice were intraperitoneally injected with TalaA. As seen in Fig. 7A, the tumor growth speed decreased after the TalaA injection was administered. The final tumor weight in the TalaA treatment group was significantly lower than that in the control group (Fig. 7B). Conversely, the treatment with TalaA affected neither mice body weight (Fig. 7C, D) nor routine blood indexes (Fig. S8), evidencing the low toxicity or side effect of TalaA. Figure 7E illustrates the histopathological staining results of our in vivo experiment, in which TalaA decreased the Ki67 level in the xenograft tumor, meaning TalaA was able to retard tumor growth. Moreover, the IHC-staining result showed that TalaA treatment decreased the level of the ferroptosis-related molecule SLC7A11 but increased that of HMOX1, which is consistent with the results obtained in our cell experiment. The H&E staining results confirmed that TalaA did not lead to the histomorphological alterations in the mice liver and kidney.

## Discussion

Different pathways of cell death exist, including apoptosis, autophagy, and necrosis^27^. Cell apoptosis induction is one of the most important therapeutic approaches for the treatment of tumors, especially in chemotherapy^16,28^. Caspase-based apoptosis has been long considered the main form of regulated cell death, which has been widely used for the development of anticancer drugs. However, treatment outcomes are usually unsatisfactory due to acquired resistance of cancer cells to apoptosis^29^. In clinical cases, the overexpression of anti-apoptotic molecules diminished the positive therapeutic outcomes against malignant cells and even aggravated the disease^30^. In recent years, the traditional understanding of regulated cell death has been challenged by the discovery of a novel cell death pathway that is distinct from apoptosis, autophagy, and necrosis^17^. Although cell ferroptosis can be distinguished from apoptosis in many ways, two major differences can be pointed out: (1) ferroptosis is directly or indirectly caused by an iron-death initiator, and lipid peroxide (LPO) severely damages cell integrity and structure^17^; (2) Ferroptosis bypasses apoptosis inhibition, avoiding the induction of membrane-specific proteins (such as P-glycoprotein and multi-resistant-related protein family) related to multidrug resistance, which may provide novel insights into the development of chemo-resistant tumor therapy^31^. Moreover, it is worth noting that many mesenchymal cancer cells, which are prone to metastasis and are usually resistant to various treatments, are sensitive to ferroptosis^32,33^. Therefore, achieving cancer cell death via ferroptosis induction could be a novel strategy for metastatic cancer treatment.

The significance of ferroptosis is remarkable because iron serves as both an acceptor and a donor of electrons; it is not only the necessary nutrients, but also the excess of toxins; it is not only the motility factor of oxidative stress, but also the braking factor of oxidative stress^34^. Homeostasis imbalance of iron not only leads to DNA oxidative damage and increased tumorigenesis, but also contributes to cancer cell apoptosis through the process of iron-induced cell death^35^. Despite the infancy of ferroptosis-related anticancer research, its importance and potential for clinical treatment are increasingly prominent.

In this study, we discovered that TalaA can kill CRC cells by ferroptosis induction. The ferroptosis inhibitor did not completely attenuate TalaA-caused cell death, which suggested that we should not exclude the likelihood that TalaA may induce cell death through other mechanisms. Nevertheless, both the lipid peroxidation inhibitor ferrostatin-1 and the iron-chelating reagent deferiprone alleviated TalaA-induced cell death, showing that ferroptosis is the main mechanism by which TalaA induces cell death. It is noteworthy that the capability of TalaA to induce ferroptosis is stronger than that of erastin, a well-known specific ferroptosis inducer^36^. Moreover, IC_50_ of TalaA was much lower than that of erastin in CRC cells, indicating that TalaA would have greater potential as a therapeutic agent in cancer treatment than erastin.

In the present study, TalaA treatment markedly decreased the level of an important channel protein in CRC cells, SLC7A11. Consistently, several recent studies reported that SLC7A11 was closely negatively correlated with ferroptosis^37^, and the suppression of SLC7A11 induced ferroptosis^38^. SLC7A11 is a trans-membrane amino-acid transporter of extracellular cystine, which is essential for glutathione synthesis, into cells^39^. In recent years, this nutrient transporter has been linked to the occurrence of a new form of iron-dependent cell death, caused by excessive iron-dependent cell accumulation of LPOs^19^. Since cystine is an essential biosynthesis precursor to glutathione, cystine depletion or SLC7A11-contained cysteine transport blockage can lead to cell ferroptosis. Interestingly, besides SLC7A11, we also found that a glutathione synthetase, GSS, was significantly downregulated in TalaA-treated CRC cells. Our discoveries evidenced that TalaA is a potent blocker of the SLC7A11–GSS–GSH axis, which is positively associated with ferroptosis. Other research has also been focused on the use of SLC7A11 as a target molecule for ferroptosis promotion^40^.

*ALOXE3*, a gene-encoding arachidonate lipoxygenase 3, is a representative of the lipoxygenase family, which catabolizes the oxidation of arachidonic acid-derived compounds^41^. In our study, both RNA-Seq and RT-qPCR results showed that TalaA upregulated *ALOXE3* expression, which indicates that TalaA might trigger lipid polyunsaturated fatty acid peroxidation via elevation of arachidonate lipoxygenase 3 levels. Lipid peroxidation has been previously reported to be a key step in ferroptosis^42^. We established that TalaA upregulated the expression of ALXOE3 and increased lipid peroxidation, which in turn enhanced ferroptosis.

We also found that TalaA considerably increased the expression of gene-encoding heme oxygenase (HMOX1), an essential enzyme in heme catabolism which cleaves heme to generate biliverdin, subsequently converted to bilirubin and carbon monoxide by biliverdin reductase^43^. Fang et al. reported that HMOX1 upregulation led to heme degradation and the release of free iron, which can be accumulated in mitochondria and cause lipid peroxidation^44^. Recently, the noncanonical ferroptosis induction function of HMOX1 has mentioned that HMOX1 activation can lead to heme degradation, in turn releasing labile Fe (II) through direct targeting of Kelch-like ECH-associated protein 1 (KEAP1), which can trigger ferroptosis^45^. The findings of our study indicated that TalaA killed CRC cells by triggering ferroptosis via acceleration of lipid peroxidation.

**Fig. 8:**
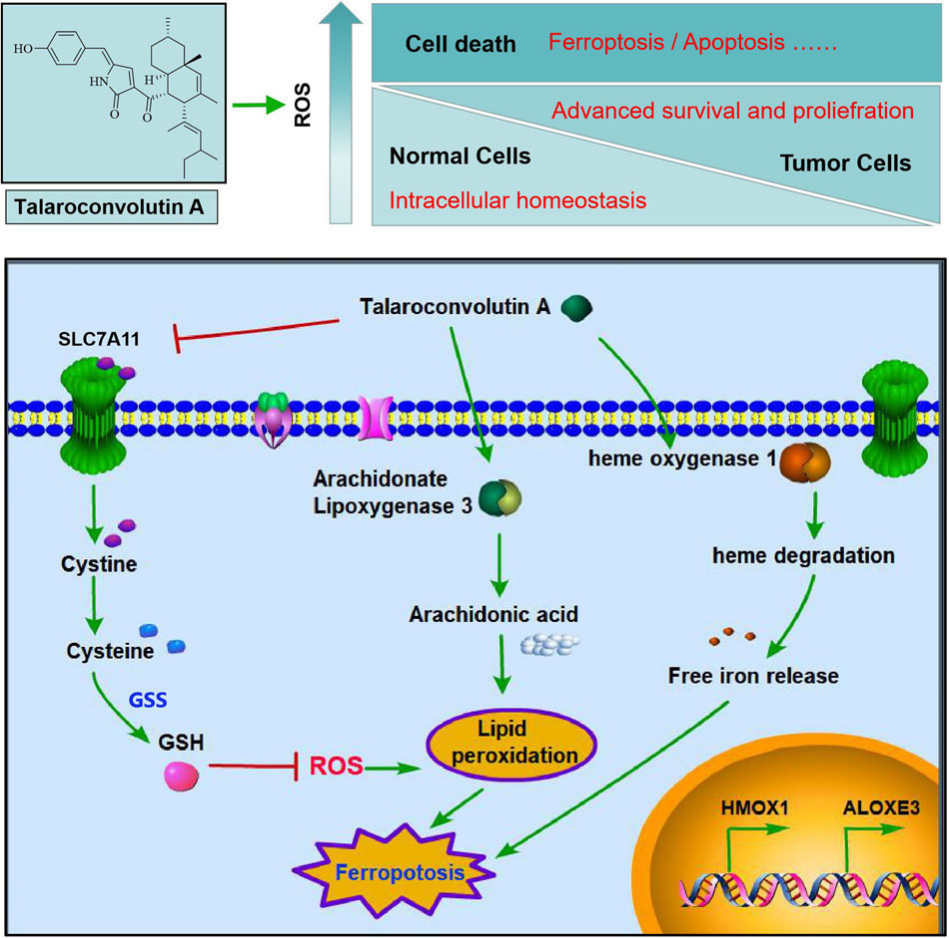
The table of content (TOC figure). In normal healthy cells the ROS is low, and REDOX reaches intracellular homeostasis; but in cancer cells, due to vigorous cell metabolism and proliferation, the ROS level is much higher. However, a set of antioxidant system against ROS is derived by tumor cells, so that tumor cells cannot be harmed by ROS, but utilize ROS as a positive regulatory signal for advanced survival and proliferation. When ROS level continues to rise beyond the tolerance threshold of tumor cells, a programmed death (such as ferroptosis) will be triggered. TalaA was able to strongly induce ferroptpsis at least via the following mechanism: (1) TalaA elevates the ROS level in colorectal cells; (2) TalaA down regulates the SLC7A11 and GSS expressions, which suppresses the synthesis of important antioxidant molecule—GSH, and in turn enhance ferroptosis; (3) oxidation of arachidonic acid is an important cause of iron death, and TalaA increases the arachidonic acid oxidase—ALOXE3, which accelerates ferroptosis. (4) TalaA causes upregulation of HMOX1 which lead to the degradation of heme and the release of free iron, accumulating in mitochondria and giving rise to lipid peroxidation.

In normal cells where a redox balance exists, ROS levels are usually low, and a variety of antioxidant substances are available to counteract the damaging effects caused by ROS. However, due to the high metabolic activity in tumor cells, excessive ROS is generated, but tumor cells are able to adjust the signaling channel to adapt to the high ROS level, including raising the expression of antioxidant molecules quantity (such as SOD, GSH, and thioredoxin), to remove excessive ROS, thus ensuring the proliferation and survival of tumor cells. Notably, in cases of continuous increased ROS level, a breakthrough is achieved upon reaching a certain threshold; thereafter, excessive oxidative stress can cause irreparable cell damage or trigger programmed cell death (e.g., ferroptosis)^25^. In other words, the baseline ROS level of tumor cells is already high, and a further increase or impaired ability to counteract ROS action can result in tumor cell death (Fig. 8). The development of antitumor drugs based on the aforementioned tumor cell features would represent an effective strategy for cancer therapy. The natural compound TalaA does exactly that: on one hand, it elevates the ROS level of cancer cells, but on the other, the altered expression of ferroptosis-related molecules accelerates cancer cell death via ferroptosis induction. Moreover, the in vivo experiments in this study showed that TalaA neither affected mice body weight and blood routine index, nor damaged the liver and kidney tissues in mice. Therefore, the potential of this valuable compound for the development of an anticancer drug is immense.

## Conclusion

The present study not only discovered a new function of TalaA—its ability to kill tumor cells via ferroptosis induction, but also elucidated its molecular pharmacological mechanism of upregulated expression of molecules (such as ALOXE3 and HMOX1), which promotes lipid peroxidation and suppresses the expression of antioxidant-related molecules (such as SLC7A11 and GSS), thereby causing cancer cell death by ferroptosis induction (Fig. 8). Nevertheless, we do not rule out the possibility of other molecular pharmacological mechanisms for achieving cancer cell death. Therefore, TalaA is a potential drug candidate that can not only take advantage of the high ROS level to kill cancer cells, but also provides targeted therapy for cancer types with high expression of anti-oxidation molecules such as SLC7A11. This study is of great significance for the development of new anticancer drugs via ferroptosis induction.

## Supplementary information

Supplementary Figure Legends

Supplementary Figure S1

Supplementary Figure S2

Supplementary Figure S3

Supplementary Figure S4

Supplementary Figure S5

Supplementary Figure S6

Supplementary Figure S7

Supplementary Figure S8
