# Supplementary material for: Discovery of a novel ferroptosis inducer-talaroconvolutin A—killing colorectal cancer cells in vitro and in vivo
Source: Cell Death Dis. 2020 Nov 17;11(11):988. doi: 10.1038/s41419-020-03194-2 (PMC7673992; doi:10.1038/s41419-020-03194-2)
Supplement: Supplementary file 1 — Supplementary Figure Legends [file 41419_2020_3194_MOESM1_ESM.docx]

**Supplementary Figure Legends**

**Figure S1.** The purity of talaroconvolutin A (TalaA) was tested by HPLC (agilent-1260). The peak appears at 16.875 min represents the purified TalaA.

**Figure S2.** Identification of TalaA by nuclear magnetic resonance (NMR). (A)^1^H-NMR spectrum of TalaA (CD3OD, 600 MHz); (B) ^13^C-NMR spectrum of TalaA (CD3OD, 150 MHz).

**Figure S3.** The chemical structure of TalaA. The numbers in figure represent the atomic order of TalaA.

**Figure S4.** TalaA suppressed colorectal cancer cells activities time-dependently. Colorectal cancer HCT116, SW480 and SW620 cells was treated by 5 μM TalaA and the relative cell activity was examined with CCK8 kit (set the cells without TalaA treatment as 100%).

**Figure S5.** The iron chelating reagent-deferiprone rescued TalaA induced cell death. (A) The SW480 cells were treated by different concentrations of deferiprone for 24 h. The CCK8 was performed to test the cell proliferation rate. The star markers indicated significant difference analyzed by t-test (* *p*<0.05) versus control (0 μM deferiprone), N=3 independent repeats. (B) The cells pre-treated by different concentration of deferiprone for 1h were incubated with 12 μM talaA for 24h. The cell proliferation rate was tested by CCK8; * *p*<0.05, ** *p*<0.01 versus TalaA treatment, N=3 independent repeats.

**Figure S6.** Ferroptosis related molecules were tested by RT-qPCR. To verify the RNA-seq results, the SW480 cells were treated by 0 μM, 5 μM and 15 μM TalaA. After the RNA was extracted and the cDNA was reverse transcripted, real-time PCR was performed. The star markers indicated significant difference analyzed by t-test (* *p*<0.05), N=3 independent repeats.

**Figure S7.** The anti-cancer activity combination of TalaA with iFSP1 and FIN56. SW480 cells were treated with talaA (4μM), FSP1 inhibitor - iFSP1 (1μM), GPX4 inhibitor - FIN56 (5μM), as well as their combination for 24 hours. The cell proliferation was detected by CCK8 kit. * *p*<0.05, ** *p*<0.01, N=3 independent repeats.

**Figure S8.** Comparison the blood routine index between TalaA treated mice group and control group. To test the toxic effects of TalaA, the erythrocyte count, white blood cell count, lymphocyte count, basophilic granulocyte count, monocytes count and blood platelet count were shown. The label “ns” means no significant different, N=6 mice.
