# Supplementary figures and images for: Discovery of a novel ferroptosis inducer-talaroconvolutin A—killing colorectal cancer cells in vitro and in vivo
Source: Cell Death Dis. 2020 Nov 17;11(11):988. doi: 10.1038/s41419-020-03194-2 (PMC7673992; doi:10.1038/s41419-020-03194-2)

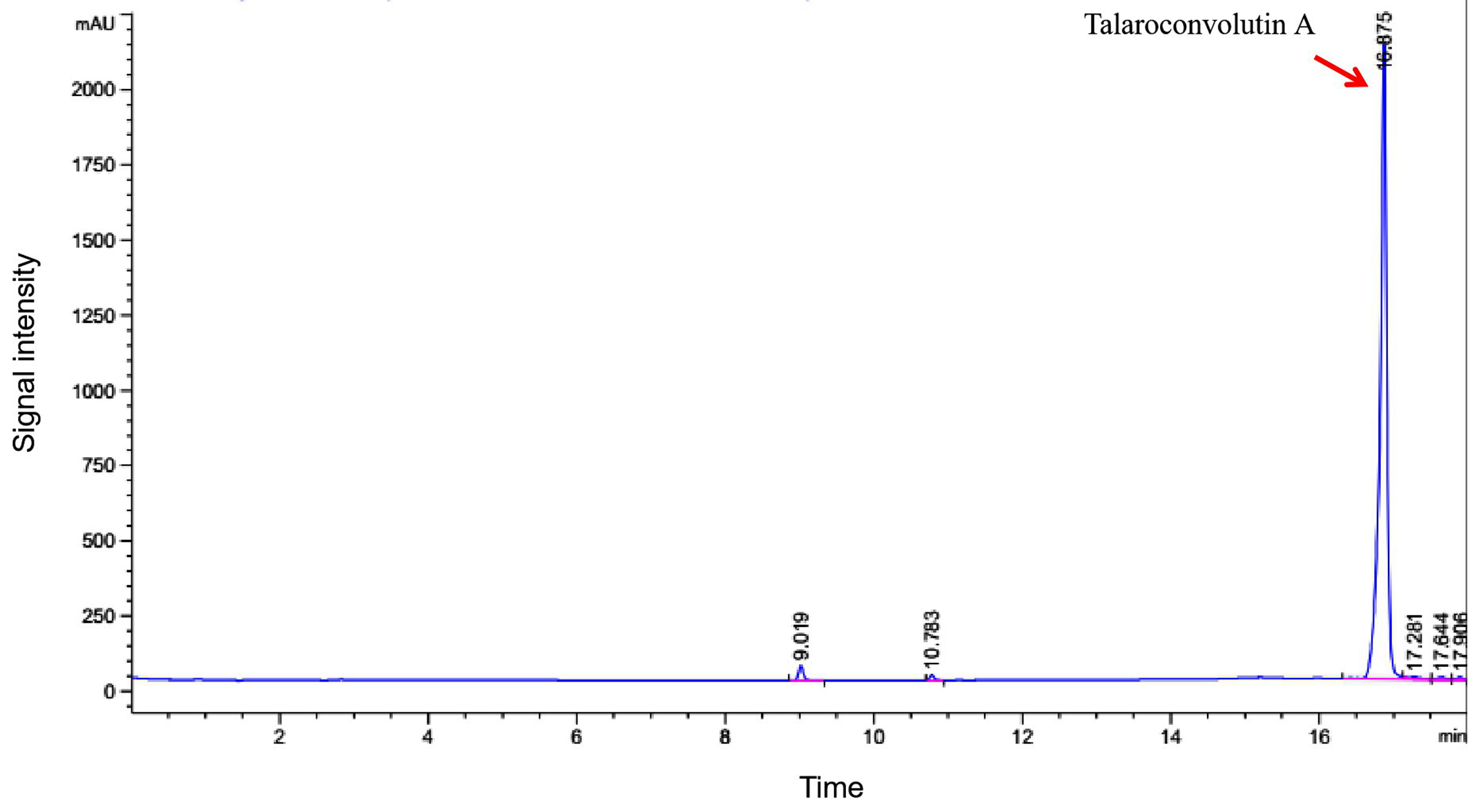

Supplement: Supplementary file 2 — Supplementary Figure S1 [file 41419_2020_3194_MOESM2_ESM.jpg]

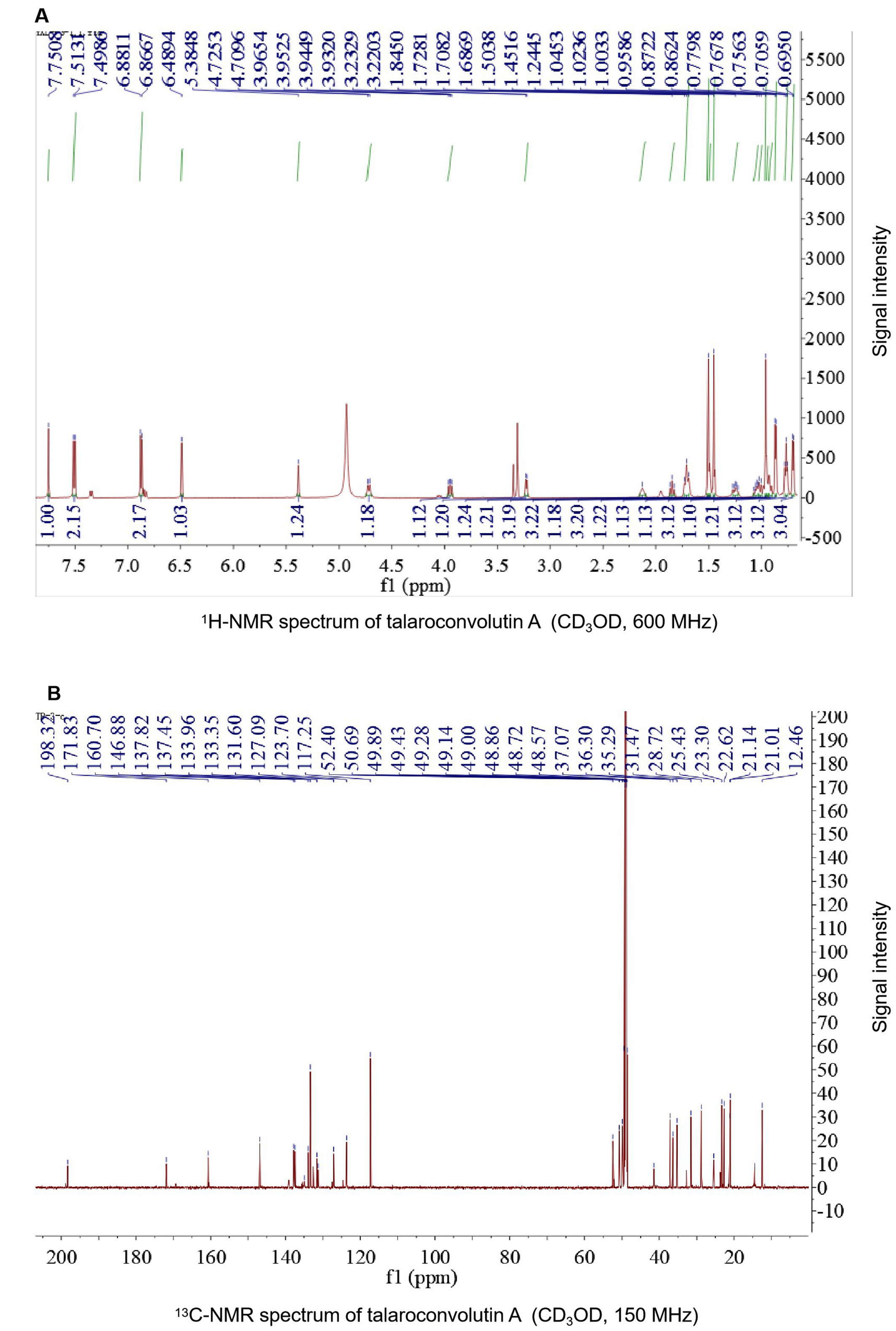

Supplement: Supplementary file 3 — Supplementary Figure S2 [file 41419_2020_3194_MOESM3_ESM.jpg]

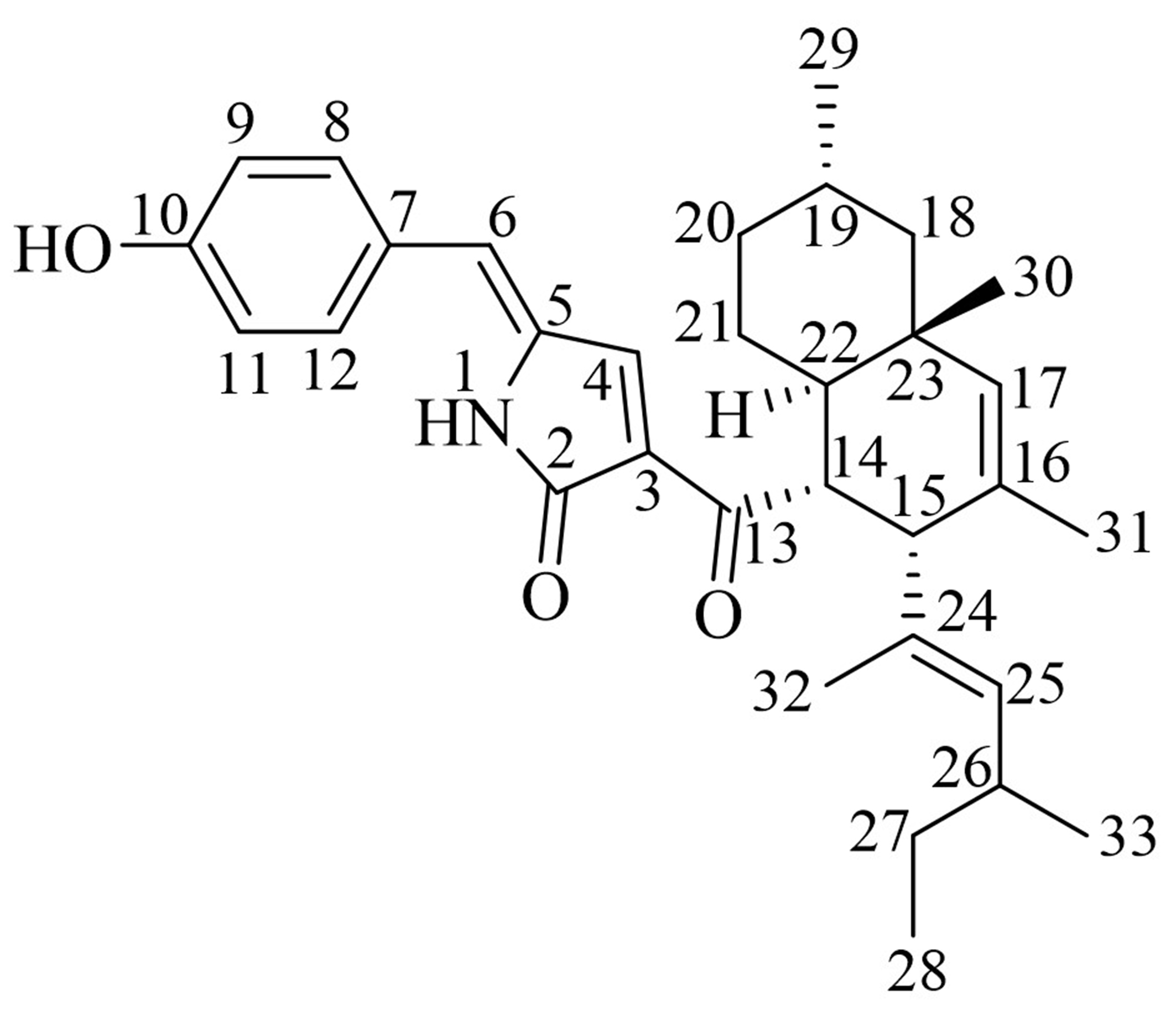

Supplement: Supplementary file 4 — Supplementary Figure S3 [file 41419_2020_3194_MOESM4_ESM.jpg]

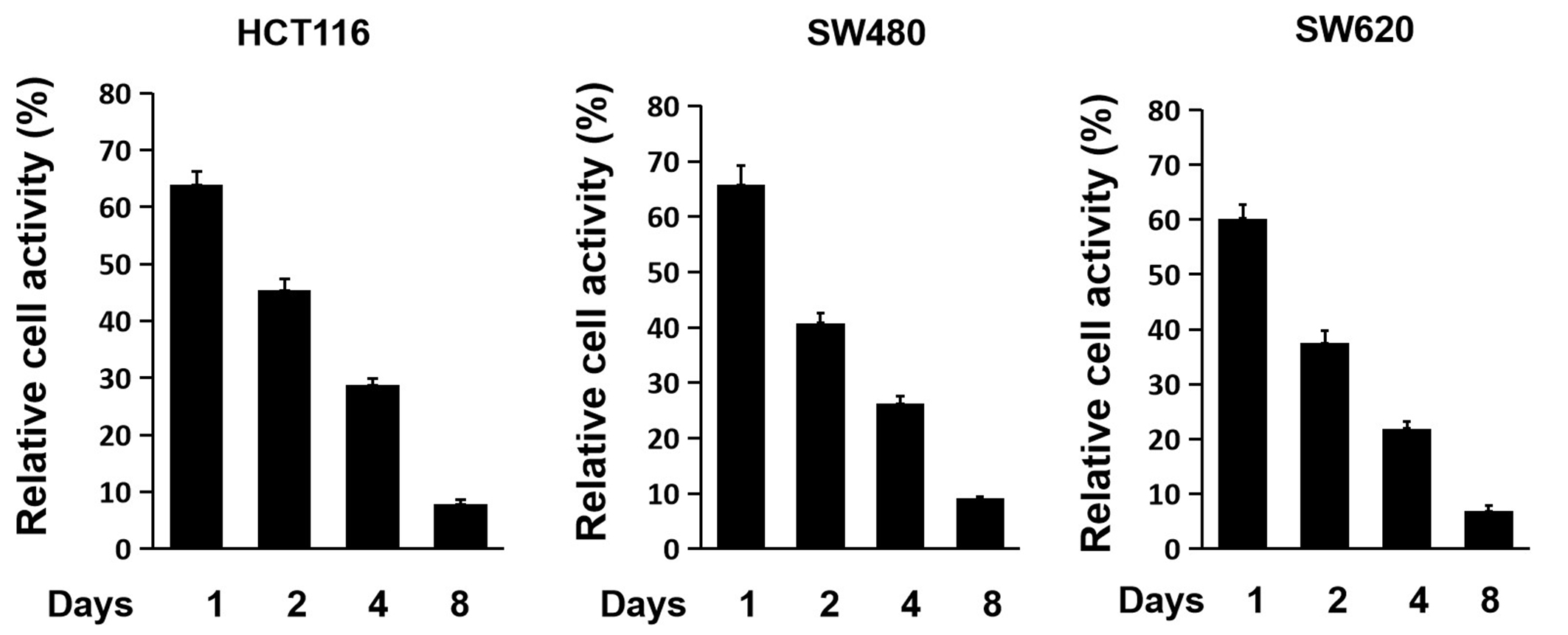

Supplement: Supplementary file 5 — Supplementary Figure S4 [file 41419_2020_3194_MOESM5_ESM.jpg]

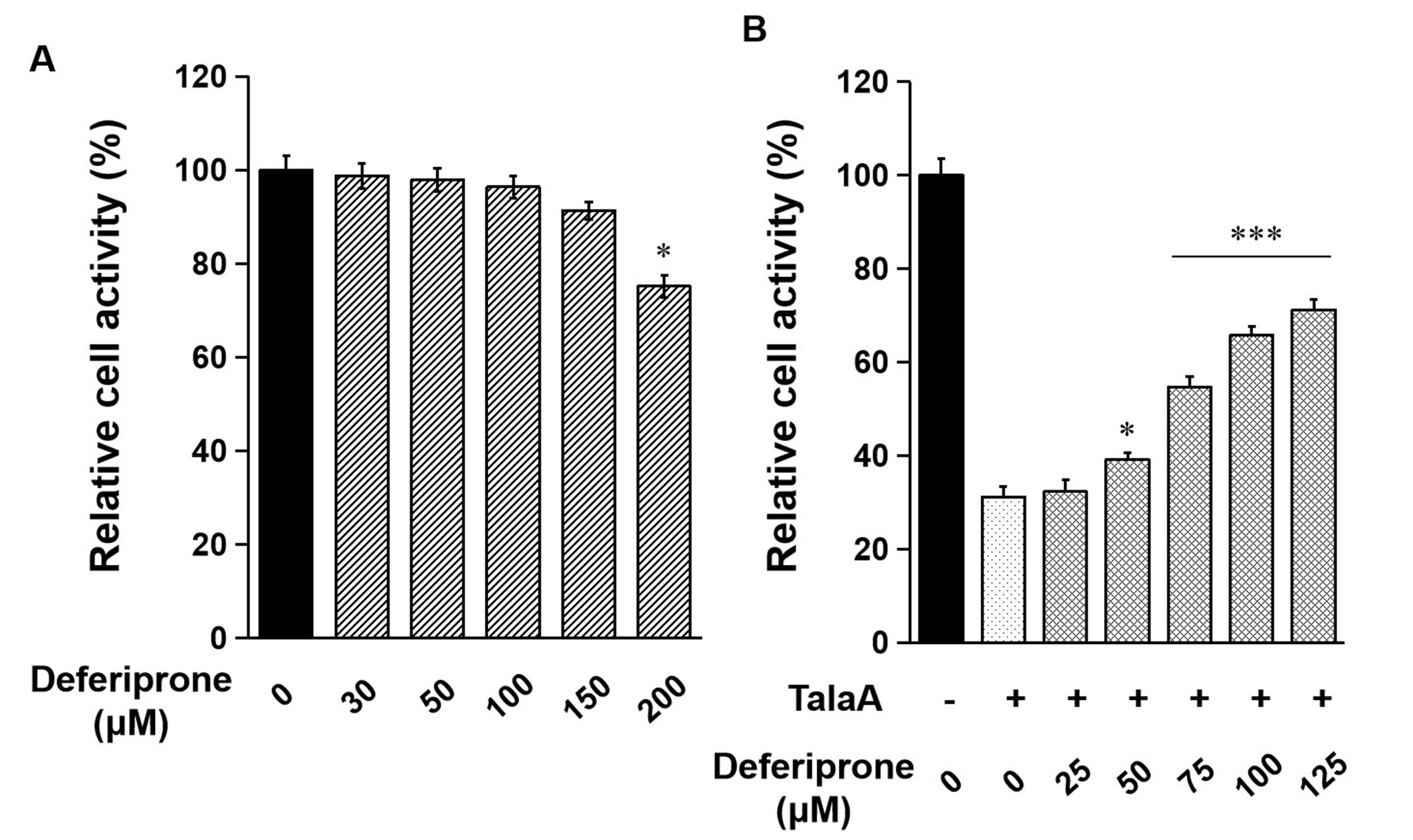

Supplement: Supplementary file 6 — Supplementary Figure S5 [file 41419_2020_3194_MOESM6_ESM.jpg]

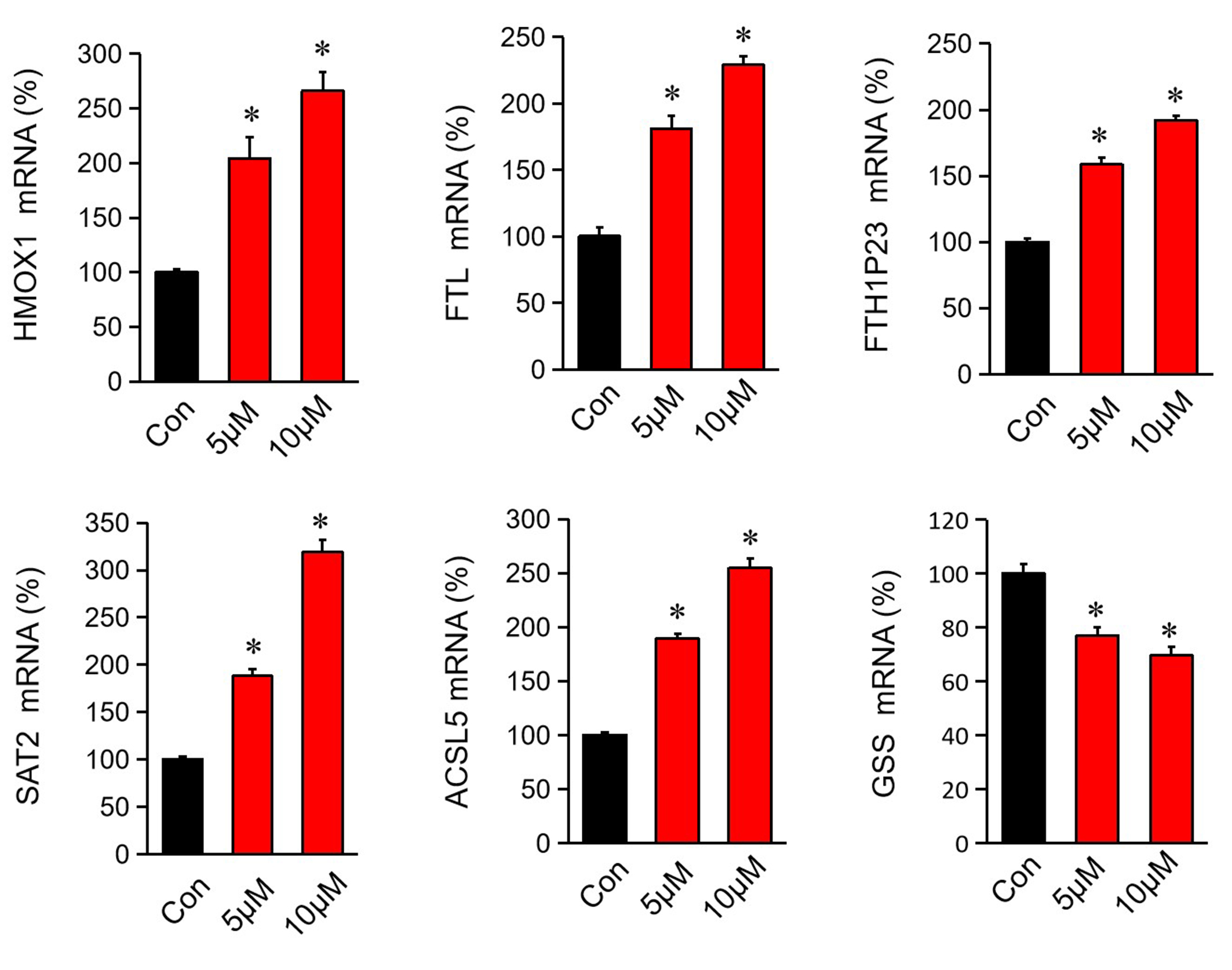

Supplement: Supplementary file 7 — Supplementary Figure S6 [file 41419_2020_3194_MOESM7_ESM.jpg]

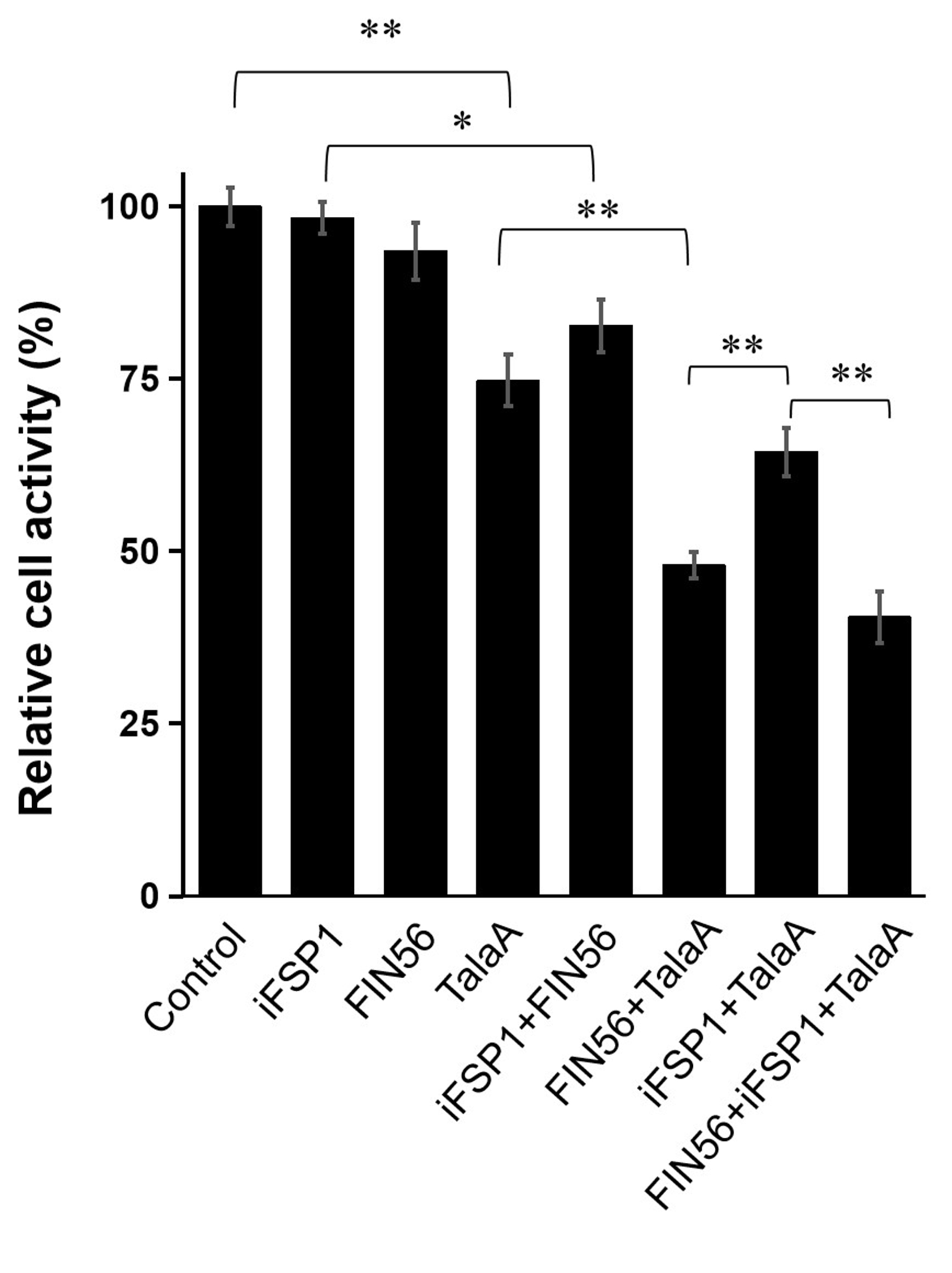

Supplement: Supplementary file 8 — Supplementary Figure S7 [file 41419_2020_3194_MOESM8_ESM.jpg]

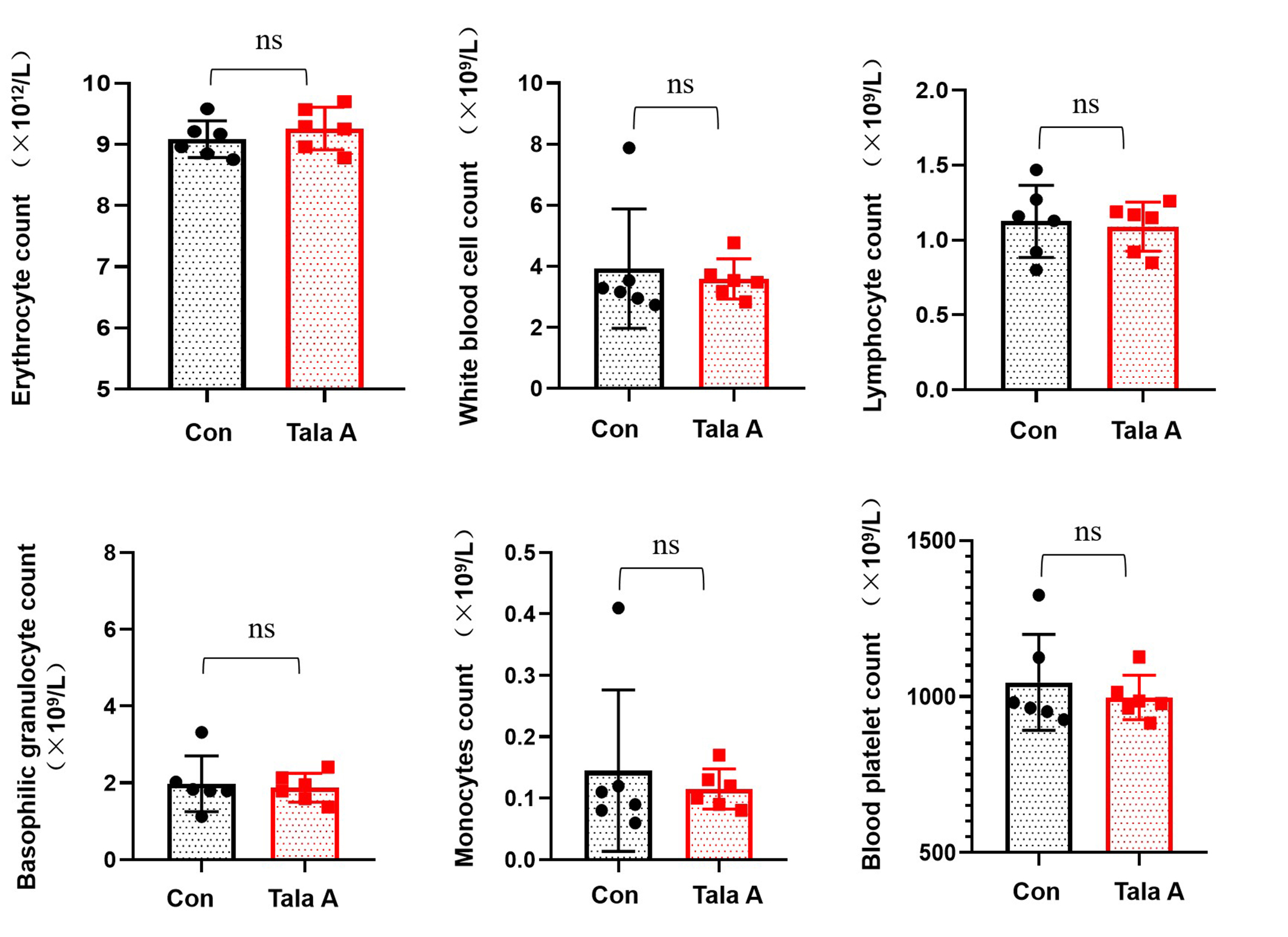

Supplement: Supplementary file 9 — Supplementary Figure S8 [file 41419_2020_3194_MOESM9_ESM.jpg]
